# Assessment of ^18^F-PI-2620 as a Biomarker in Progressive Supranuclear Palsy

**DOI:** 10.1001/jamaneurol.2020.2526

**Published:** 2020-07-07

**Authors:** Matthias Brendel, Henryk Barthel, Thilo van Eimeren, Ken Marek, Leonie Beyer, Mengmeng Song, Carla Palleis, Mona Gehmeyr, Urban Fietzek, Gesine Respondek, Julia Sauerbeck, Alexander Nitschmann, Christian Zach, Jochen Hammes, Michael T. Barbe, Oezguer Onur, Frank Jessen, Dorothee Saur, Matthias L. Schroeter, Jost-Julian Rumpf, Michael Rullmann, Andreas Schildan, Marianne Patt, Bernd Neumaier, Olivier Barret, Jennifer Madonia, David S. Russell, Andrew Stephens, Sigrun Roeber, Jochen Herms, Kai Bötzel, Joseph Classen, Peter Bartenstein, Victor Villemagne, Johannes Levin, Günter U. Höglinger, Alexander Drzezga, John Seibyl, Osama Sabri

**Affiliations:** 1 Department of Nuclear Medicine, University Hospital of Munich, LMU Munich, Munich, Germany; 2 Department of Nuclear Medicine, University of Leipzig, Leipzig, Germany; 3 Department of Nuclear Medicine, University Hospital Cologne, Cologne, Germany; 4 Department of Neurology, University Hospital Cologne, Cologne, Germany; 5 German Center for Neurodegenerative Diseases (DZNE), Bonn-Cologne, Germany; 6 InviCRO LLC, Boston, Massachusetts; 7 Molecular Neuroimaging, A Division of InviCRO, New Haven, Connecticut; 8 Department of Neurology, University Hospital of Munich, LMU Munich, Munich, Germany; 9 Department of Neurology, Hannover Medical School, Hannover, Germany; 10 Department of Psychiatry, University Hospital Cologne, Cologne, Germany; 11 Center for Memory Disorders, University Hospital Cologne, Cologne, Germany; 12 Department of Neurology, University of Leipzig, Leipzig, Germany; 13 Clinic for Cognitive Neurology, University of Leipzig, Leipzig, Germany; 14 LIFE–Leipzig Research Center for Civilization Diseases, University of Leipzig, Leipzig, Germany; 15 Max Planck Institute for Human Cognitive and Brain Sciences, Leipzig, Germany; 16 Forschungszentrum Jülich GmbH, Institute of Neuroscience and Medicine, Nuclear Chemistry (INM-5), Jülich, Germany; 17 Life Molecular Imaging GmbH, Berlin, Germany; 18 Center for Neuropathology and Prion Research, University Hospital of Munich, LMU Munich, Munich, Germany; 19 German Center for Neurodegenerative Diseases (DZNE), Munich, Germany; 20 Munich Cluster for Systems Neurology (SyNergy), Munich, Germany; 21 Department of Molecular Imaging & Therapy, Austin Health, Heidelberg, Victoria, Australia; 22 Department of Medicine, Austin Health, The University of Melbourne, Melbourne, Victoria, Australia; 23 Department of Neurology, Technical University Munich, Munich, Germany

## Abstract

**Importance:**

Progressive supranuclear palsy (PSP) is a 4-repeat tauopathy. Region-specific tau aggregates establish the neuropathologic diagnosis of definite PSP post mortem. Future interventional trials against tau in PSP would strongly benefit from biomarkers that support diagnosis.

**Objective:**

To investigate the potential of the novel tau radiotracer ^18^F-PI-2620 as a biomarker in patients with clinically diagnosed PSP.

**Design, Setting, and Participants:**

In this cross-sectional study, participants underwent dynamic ^18^F-PI-2620 positron emission tomography (PET) from 0 to 60 minutes after injection at 5 different centers (3 in Germany, 1 in the US, and 1 in Australia). Patients with PSP (including those with Richardson syndrome [RS]) according to Movement Disorder Society PSP criteria were examined together with healthy controls and controls with disease. Four additionally referred individuals with PSP-RS and 2 with PSP–non-RS were excluded from final data analysis owing to incomplete dynamic PET scans. Data were collected from December 2016 to October 2019 and were analyzed from December 2018 to December 2019.

**Main Outcomes and Measures:**

Postmortem autoradiography was performed in independent PSP-RS and healthy control samples. By in vivo PET imaging, ^18^F-PI-2620 distribution volume ratios were obtained in globus pallidus internus and externus, putamen, subthalamic nucleus, substantia nigra, dorsal midbrain, dentate nucleus, dorsolateral, and medial prefrontal cortex. PET data were compared between patients with PSP and control groups and were corrected for center, age, and sex.

**Results:**

Of 60 patients with PSP, 40 (66.7%) had RS (22 men [55.0%]; mean [SD] age, 71 [6] years; mean [SD] PSP rating scale score, 38 [15]; score range, 13-71) and 20 (33.3%) had PSP–non-RS (11 men [55.0%]; mean [SD] age, 71 [9] years; mean [SD] PSP rating scale score, 24 [11]; score range, 11-41). Ten healthy controls (2 men; mean [SD] age, 67 [7] years) and 20 controls with disease (of 10 [50.0%] with Parkinson disease and multiple system atrophy, 7 were men; mean [SD] age, 61 [8] years; of 10 [50.0%] with Alzheimer disease, 5 were men; mean [SD] age, 69 [10] years). Postmortem autoradiography showed blockable ^18^F-PI-2620 binding in patients with PSP and no binding in healthy controls. The in vivo findings from the first large-scale observational study in PSP with ^18^F-PI-2620 indicated significant elevation of tracer binding in PSP target regions with strongest differences in PSP vs control groups in the globus pallidus internus (mean [SD] distribution volume ratios: PSP-RS, 1.21 [0.10]; PSP–non-RS, 1.12 [0.11]; healthy controls, 1.00 [0.08]; Parkinson disease/multiple system atrophy, 1.03 [0.05]; Alzheimer disease, 1.08 [0.06]). Sensitivity and specificity for detection of PSP-RS vs any control group were 85% and 77%, respectively, when using classification by at least 1 positive target region.

**Conclusions and Relevance:**

This multicenter evaluation indicates a value of ^18^F-PI-2620 to differentiate suspected patients with PSP, potentially facilitating more reliable diagnosis of PSP.

## Key Points

**Question:**

Can tau–positron emission tomography imaging with the novel tau radiotracer ^18^F-PI-2620 differentiate patients with progressive supranuclear palsy (PSP) from healthy controls and controls with disease?

**Findings:**

In this cross-sectional study of 60 patients with PSP, 10 healthy controls, and 20 controls with disease, there was significantly higher ^18^F-PI-2620 binding in target regions of patients with PSP compared with controls regardless of disease severity. Individual patients with PSP with Richardson syndrome were separated with high sensitivity and specificity.

**Meaning:**

^18^F-PI-2620 tau–positron emission tomography differentiates patients with PSP from controls at the single-patient level, potentially facilitating a more reliable diagnosis.

## Teaser

This cross-sectional study investigates the potential of novel tau radiotracer ^18^F-PI-2620 as a biomarker in patients with clinically diagnosed progressive supranuclear palsy.

## Introduction

Progressive supranuclear palsy (PSP), initially described by Steele et al,^1^ is a primary 4-repeat tauopathy^2^ clinically characterized by postural instability with falls and impaired volitional eye movements and leading to death with a median of 8 years after symptom onset.^2^ However, clinical symptoms and subtypes of PSP also have strong overlaps with other neurodegenerative disorders such as TDP-43–positive frontotemporal dementia or corticobasal degeneration.^3,4^ Thus, clinical assessments in PSP are lacking sensitivity early in the disease course and have a limited specificity for the pathologic entity.^5^ Therefore, biomarkers for PSP have a great range of potential utility, for which formal criteria are now available.^6^ Current PSP diagnosis criteria already take imaging of atrophy or hypometabolism by magnetic resonance imaging or positron emission tomography (PET) into account.^5^ However, no available biomarker currently fulfills the criteria for an ideal biomarker, which would be positive in a presymptomatic stage, specific for any variant of pathology, and anticipate disease progression.^6^ As more established readouts (hypometabolism, atrophy) represent late events in the disease course, tau-PET imaging may be able to provide more favorable biomarker information for early detection of PSP. Other tau-PET tracers were reported to be able to differentiate patients with PSP from controls,^7,8,9^ but the observed binding was not confirmed to specifically relate to tau.^10,11,12^ The novel tau-PET tracer ^18^F-PI-2620 proved absent off-target binding to monoamine oxidases,^13^ high affinity to 3/4R tau in Alzheimer disease (AD),^14^ but also high affinity to recombinant 4R tau fibrils and PSP brain homogenate,^13^ and colocalized binding to proven 4R tau by a combination of micro-autoradiography and immunohistochemistry in PSP tissue.^13^

The main aim of this multicenter evaluation was to investigate the diagnostic capability of ^18^F-PI-2620 PET imaging in a cohort of patients with clinically diagnosed PSP in vivo. We endeavored to test if patients with PSP can be differentiated from healthy controls and controls with disease by quantitative and visual PET image analyses. Furthermore, we performed postmortem autoradiography from independent brain bank samples to test if the ^18^F-PI-2620 signal in the basal ganglia and the frontal cortex of PSP can be distinguished and blocked by nonlabeled compound.

## Methods

### Postmortem Brain Tissue Analyses

Samples from 4 deceased patients with PSP with Richardson syndrome (RS) and those from 4 deceased healthy controls, which were independent from the in vivo cohort, were analyzed by immunohistochemistry and ^18^F-PI-2620 in vitro autoradiography (2 individuals with PSP-RS and healthy controls each for basal ganglia and frontal cortex evaluation). Autoradiography procedures and more detailed information on cases are provided in the eMethods in the Supplement. Positron emission tomography data analyses and in vitro analyses on human brain samples were approved by the institutional ethics committee at the University Hospital of Munich, LMU Munich, in Munich, Germany. All participants provided written informed consent prior to the PET scan. The observational study was registered at the German Clinical Trials Register (DRKS00016920). Clinical data were collected according to a standardized protocol via the German multicenter prospective ProPSP cohort study.

### Investigated Population and Clinical Assessments

Patients with probable or possible PSP according to current diagnosis criteria^5^ were examined together with controls with disease and healthy controls at a total of 5 different specialized centers between December 2016 and October 2019. The PSP cohort consisted of patients with PSP-RS and non–RS-variant PSP. The PSP–non-RS group consisted of individuals with predominant corticobasal syndrome, predominant frontal presentation, predominant parkinsonism, predominant speech/language disorder, and progressive gait freezing. Controls with disease were categorized either into suspected α-synucleinopathies or the AD continuum. α-Synucleinopathy controls with disease all had a probable clinical diagnosis^15,16^ and consisted of individuals with Parkinson disease and multiple system atrophy. Controls with AD continuum all had a positive β-amyloid PET (^18^F-florbetaben or ^18^F-flutemetamol), fulfilled criteria for typical AD,^17^ and were composed of individuals with mild cognitive impairment and with dementia. Some individuals of this group had early onset (age <65 years), and others had late-onset disease. Disease duration was defined as the time between symptom onset and PET imaging. The PSP rating scale served as disease severity parameter for the included patients with PSP, and the Montreal Cognitive Assessment (MoCA) or converted Mini-Mental State Examination scores^18^ served as a cognition deficit severity parameter. Schwab and England Activities of Daily Living scores were recorded as a global score of function ability.

### PET Imaging

^18^F-PI-2620 PET imaging was performed in a full dynamic setting (0-60 minutes postinjection) at 5 different neuroimaging sites using PET/computed tomography or PET/magnetic resonance imaging systems. Details of radiosynthesis, PET acquisition, reconstruction, framing, image harmonization across scanners, and spatial normalization are provided in the eMethods in the Supplement. The multilinear reference tissue model 2^19^ in PMOD version 3.9 (PMOD Inc) was used to calculate distribution volume ratio images (DVR; DVR = nondisplaceable binding potential + 1) of each full dynamic data set. The cerebellum excluding the dentate nucleus and the central cerebellar white matter as well as the superior and the posterior cerebellar layers (thickness in *z* direction = 1.5 cm each) served as a reference region (eFigure 1 in the Supplement). The clearance rate of the tracer from the reference tissue (k2′) was estimated by parallel running of multilinear reference tissue model.

### PET Data Analysis

^18^F-PI-2620 DVR values were obtained in 9 PSP target regions in the Montreal Neurology Institute space, predefined by the atlas of the basal ganglia,^20^ the Brainnetome atlas,^21^ and the Hammers atlas,^22^ based on earlier autopsy data^23^: globus pallidus (internus and externus), putamen, subthalamic nucleus, substantia nigra, dorsal midbrain, dentate nucleus, dorsolateral prefrontal cortex, and medial prefrontal cortex (eFigure 1 in the Supplement). Additionally, target and reference region values were extracted in single frames to allow calculation of standardized uptake value ratios (SUVr) during the scan time. Thirty- to 60-minute SUVr values were calculated as static ^18^F-PI-2620 uptake. A dichotomous visual read of DVR maps was performed by 3 expert readers (M.B., H.B., and T.v.E.), as described in the eMethods and in eFigure 2 in the Supplement.

### Statistics

SPSS version 25 (IBM) was used for statistical testing. Autoradiography quantification (minimum of 4 slices per sample) of patients with PSP and controls was compared one by one using an unpaired *t* test. Demographics were compared between groups by a 1-way analysis of variance. ^18^F-PI-2620 DVR values of the target regions were compared between the 5 study groups by a multivariate analysis of variance including age, sex, and center as covariates as well as post hoc Bonferroni correction for multiple group comparisons. Effect sizes (Cohen *d*) were calculated between PSP and control groups. Exploratory comparison of regional DVR *z* scores (*z* Score = [Single DVR of Patient – Mean Value of DVR of Healthy Controls] / SD of DVR of Healthy Controls) between different PSP phenotypes was performed by multivariate analysis of variance (controlled for age, sex, and center) without adjustment for multiple group comparisons. Spearman coefficients of correlation (*r*) were calculated for DVR vs age, PSP rating scale, and disease duration. Pearson coefficient of correlation (*R*) was calculated for DVR and 30- to 60-minute SUVr. *P* values less than .05 were considered significant. For semiquantitative analyses, a regional DVR greater than or equal to the mean value plus 2 SDs of the healthy controls was defined as positive. Here, 1 positive target region defined the participant as positive (dichotomous) for a PSP-like ^18^F-PI-2620 PET scan.

## Results

### Postmortem Autoradiography 

**Figure 1.**
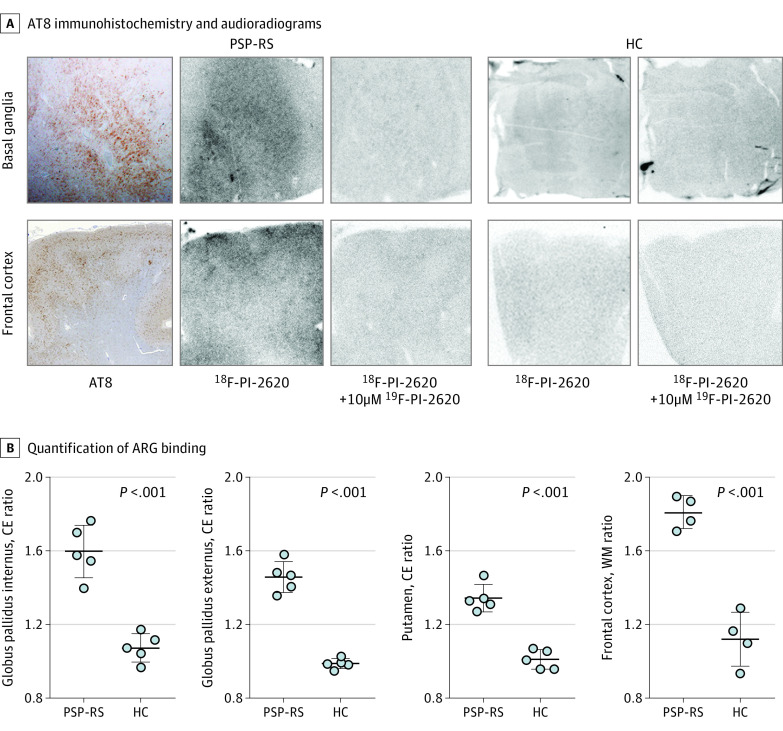
In Vitro Evaluation of ^18^F-PI-2620 Binding in Postmortem Tissue A, The top row depicts AT8 immunohistochemistry together with autoradiograms of basal ganglia slices of a man in his late 60s with a postmortem diagnosis of progressive supranuclear palsy with Richardson syndrome (PSP-RS) after incubation with ^18^F-PI-2620 alone or with ^18^F-PI-2620 and excessive cold compound (^19^F-PI-2620) as well as autoradiograms of basal ganglia slices of a healthy female control in her early 60s. The lower row depicts AT8 immunohistochemistry together with autoradiograms of frontal cortex slices of a woman in her late 60s with a postmortem diagnosis of PSP-RS after incubation with ^18^F-PI-2620 or ^18^F-PI-2620 and excessive cold compound (^19^F-PI-2620) as well as autoradiograms of frontal cortex slices of a healthy male control in his late 30s. B, Quantification of ARG binding by region of interest analysis (basal ganglia: target-to-capsula-externa [CE] ratios; frontal cortex: target-to-white matter [WM] ratios). Four or 5 brain slices of each PSP-RS and healthy control (HC) sample were analyzed and the resulting data were compared by a *t *test. Confirmatory samples are shown in eFigure 3 in the Supplement. Patient details are provided in the eMethods in the Supplement.

The unblocked basal ganglia and the frontal cortex of individuals with PSP-RS revealed a visually distinguishable ^18^F-PI-2620 binding, whereas no binding was observed in healthy controls and after blocking with excessive nonlabeled ^19^F-PI-2620 in PSP-RS (Figure 1A). The ^18^F-PI-2620 signal was colocalized with AT8-positive aggregated tau (Figure 1A), morphologically attributed to neuronal tau, tufted astrocytes, and coiled bodies.

Quantification revealed increased binding in pairs of individuals with PSP and healthy controls (mean [SD]: globus pallidus internus, 1.60 [0.14] vs 1.07 [0.08]; globus pallidus externus, 1.46 [0.08] vs 0.99 [0.03]; putamen, 1.34 [0.07] vs 1.01 [0.05]; frontal cortex, 1.81 [0.09] vs 1.12 [0.15]; Figure 1B*)*, confirmed by repetition in the second pair (mean [SD]: globus pallidus internus, 1.33 [0.07] vs 0.92 [0.15]; globus pallidus externus, 1.33 [0.11] vs 0.95 [0.13]; putamen, 1.20 [0.04] vs 1.01 [0.07]; frontal cortex, 1.28 [0.12] vs 1.07 [0.14]; eFigure 3 in the Supplement).

### Demographics of the In Vivo PET Imaging Population

Of 60 patients with PSP, 40 (66.7%) had RS (22 men [55.0%]; mean [SD] age, 71 [6] years; mean [SD] PSP rating scale score, 38 [15]; score range, 13-71) and 20 had PSP–non-RS (11 men [55.0%]; mean [SD] age, 71 [9] years; mean [SD] PSP rating scale score, 24 [11]; score range, 11-41). Ten healthy controls (2 men; mean [SD] age, 67 [7] years) and 20 controls with disease (of 10 [50.0%] with Parkinson disease and multiple system atrophy, 7 were men; mean [SD] age, 61 [8] years; of 10 [50.0%] with AD, 5 were men; mean [SD] age, 69 [10] years). Demographics and clinical scores of the study cohort are reported in the Table and specific information on all subgroups is provided in an extended version (eTable in the Supplement). There was a significant difference in age, indicating that the probable patients with α-synucleinopathy had a lower age (mean [SD], 61 [8] years) compared with both PSP groups (mean [SD] age for individuals with RS: 71 [6] years; mean [SD] age for individuals with non-RS: 71 [9] years). Cognition was significantly different, with the AD-continuum patients yielding a worse cognitive performance (mean [SD] MoCA score, 15.6 [7.8]) compared with individuals with PSP–non-RS (mean [SD] MoCA score, 23.1 [3.9]), α-synucleinopathies (mean [SD] MoCA score, 25.6 [4.1]), and healthy controls (mean [SD] MoCA score, 28.8 [1.6]) but without indicating a significant different cognition compared with PSP-RS (mean [SD] MoCA score, 20.7 [7.5]).

**Table.** Demographics and Quantitative PET Results at the Group Level^a^

Demographic	PSP-RS	PSP–non-RS	α-Synucleinopathies	Individuals with AD	Healthy controls
No.	40	20	10	10	10
Subgroups	NA	PSP-CBS (n = 9), PSP-F (n = 5), PSP-P (n = 4), PSP-SL (n = 1), PSP-PGF (n = 1)	PD (n = 6), MSA (n = 4)	MCI (n = 2), dementia (n = 8)	NA
Age, mean (SD), y	71 (6)	71 (9)	61 (8)	69 (10)	67 (7)
Sex, No. (%)					
Female	18 (45)	9 (45)	3 (30)	5 (50)	8 (80)
Male	22 (55)	11 (55)	7 (70)	5 (50)	2 (20)
Scan site center	MUC (n = 21); LPZ (n = 11); COL (n = 3); MNI (n = 4)	MUC (n = 16); LPZ (n = 4)	MUC (n = 10)	MUC (n = 6); MNI (n = 4)	MNI (n = 5); AUS (n = 5)
PSP rating scale score, mean (SD)	37.2 (15.1)	26.2 (9.6)	NA	NA	NA
PSP, No. (%)					
Possible	6 (15)	12 (60)	NA	NA	NA
Probable	34 (85)	8 (40)	NA	NA	NA
Hoehn and Yahr Scale score, mean (SD)	NA	NA	2.4 (0.8)	NA	NA
UPDRS score, mean (SD)	NA	NA	23.9 (6.2)	NA	NA
Disease duration, mean (SD), mo	49 (38)	42 (37)	20 (17)	28 (29)	NA
MoCA score, mean (SD)	20.7 (7.5)	23.1 (3.9)	25.6 (4.1)	15.6 (7.8)	28.8 (1.6)
SEADL score, mean (SD)	55 (21)	65 (17)	NA	NA	NA
**Regional PET results**					
Globus pallidus externus					
Mean (SD)	1.16 (0.10)	1.10 (0.11)	1.01 (0.06)	1.05 (0.06)	0.99 (0.05)
Cohen *d*					
Probable α-synucleinopathies	1.83	0.94	NA	NA	NA
AD	1.28	0.47	NA	NA	NA
Healthy controls	2.13	1.20	NA	NA	NA
*P* value					
Probable α-synucleinopathies	.03	>.99	NA	NA	NA
AD	.01	>.99	NA	NA	NA
Healthy controls	<.001	.02	NA	NA	NA
Globus pallidus internus					
Mean (SD)	1.21 (0.10)	1.12 (0.11)	1.03 (0.05)	1.08 (0.06)	1.00 (0.08)
Cohen *d*					
Probable α-synucleinopathies	2.23	1.08	NA	NA	NA
AD	1.49	0.45	NA	NA	NA
Healthy controls	2.28	1.27	NA	NA	NA
*P* value					
Probable α-synucleinopathies	.005	>.99	NA	NA	NA
AD	.002	>.99	NA	NA	NA
Healthy controls	<.001	.009	NA	NA	NA
Putamen					
Mean (SD)	1.19 (0.10)	1.14 (0.12)	1.05 (0.06)	1.10 (0.05)	1.02 (0.06)
Cohen *d*					
Probable α-synucleinopathies	1.65	0.93	NA	NA	NA
AD	1.12	0.43	NA	NA	NA
Healthy controls	2.04	1.26	NA	NA	NA
*P* value					
Probable α-synucleinopathies	.13	>.99	NA	NA	NA
AD	.13	>.99	NA	NA	NA
Healthy controls	.002	.10	NA	NA	NA
Subthalamic nucleus					
Mean (SD)	1.21 (0.08)	1.15 (0.09)	1.09 (0.06)	1.10 (0.08)	1.04 (0.09)
Cohen *d*	1.67	0.80	NA	NA	NA
Probable α-synucleinopathies	1.37	0.59	NA	NA	NA
AD	2.02	1.26	NA	NA	NA
Healthy controls					
*P* value	.06	>.99	NA	NA	NA
Probable α-synucleinopathies	.003	.64	NA	NA	NA
AD	<.001	.005	NA	NA	NA
Substantia nigra					
Mean (SD)	1.17 (0.09)	1.13 (0.09)	1.09 (0.06)	1.12 (0.08)	1.10 (0.07)
Cohen *d*					
Probable α-synucleinopathies	1.08	0.50	NA	NA	NA
AD	0.64	0.14	NA	NA	NA
Healthy controls	0.89	0.35	NA	NA	NA
*P* value					
Probable α-synucleinopathies	>.99	>.99	NA	NA	NA
AD	.70	>.99	NA	NA	NA
Healthy controls	.04	.53	NA	NA	NA
Dorsal midbrain					
Mean (SD)	0.87 (0.11)	0.89 (0.09)	0.92 (0.07)	0.93 (0.09)	0.92 (0.10)
Cohen *d*					
Probable α-synucleinopathies	−0.50	−0.32	NA	NA	NA
AD	−0.58	−0.43	NA	NA	NA
Healthy controls	−0.54	−0.28	NA	NA	NA
*P* value					
Probable α-synucleinopathies	>.99	>.99	NA	NA	NA
AD	>.99	>.99	NA	NA	NA
Healthy controls	>.99	>.99	NA	NA	NA
Dentate nucleus					
Mean (SD)	1.13 (0.05)	1.11 (0.05)	1.07 (0.05)	1.08 (0.03)	1.06 (0.04)
Cohen *d*					
Probable α-synucleinopathies	1.26	0.84	NA	NA	NA
AD	1.17	0.67	NA	NA	NA
Healthy controls	1.68	1.18	NA	NA	NA
*P* value					
Probable α-synucleinopathies	.13	>.99	NA	NA	NA
AD	.08	>.99	NA	NA	NA
Healthy controls	.03	.41	NA	NA	NA
Dorsolateral prefrontal cortex					
Mean (SD)	0.89 (0.09)	0.91 (0.06)	0.91 (0.05)	1.11 (0.24)	0.86 (0.08)
Cohen *d*					
Probable α-synucleinopathies	−0.28	0.09	NA	NA	NA
AD	−1.24	−1.13	NA	NA	NA
Healthy controls	0.28	0.70	NA	NA	NA
*P* value					
Probable α-synucleinopathies	>.99	>.99	NA	NA	NA
AD	<.001	<.001	NA	NA	NA
Healthy controls	>.99	>.99	NA	NA	NA
Medial prefrontal cortex					
Mean (SD)	0.83 (0.09)	0.86 (0.09)	0.89 (0.06)	0.98 (0.12)	0.89 (0.08)
Cohen *d*					
Probable α-synucleinopathies	−0.77	−0.41	NA	NA	NA
AD	−1.39	−1.14	NA	NA	NA
Healthy controls	−0.74	−0.42	NA	NA	NA
*P* value					
Probable α-synucleinopathies	.91	>.99	NA	NA	NA
AD	<.001	.008	NA	NA	NA
Healthy controls	>.99	>.99	NA	NA	NA

Abbreviations: AD, Alzheimer disease; AUS, Melbourne, Australia; COL, Cologne, Germany; LPZ, Leipzig, Germany; MCI, mild cognitive impairment; MNI, New Haven, Connecticut; MoCA, Montreal Cognitive Assessment; MSA, multiple system atrophy; MUC, Munich, Germany; MV, mean value; NA, not applicable; PD, Parkinson disease; PET, positron emission tomography; PSP, progressive supranuclear palsy; PSP-CBS, PSP with predominant corticobasal syndrome; PSP-F, PSP with predominant frontal presentation; PSP-P, PSP with predominant parkinsonism; PSP-PGF, PSP with predominant gait freezing; PSP-SL, PSP with predominant speech/language disorder; RS, Richardson syndrome; SEADL, Schwab and England Activities of Daily Living; UPDRS, Unified Parkinson Disease Rating Scale.

^a^ *P* values were derived from multivariate analysis of variance including center, age, and sex as covariates and Bonferroni adjustment for multiple comparisons of study groups. Effect sizes were calculated as Cohen *d* for both PSP study groups against different control groups.

### ^18^F-PI-2620 Binding In Vivo 

**Figure 2.**
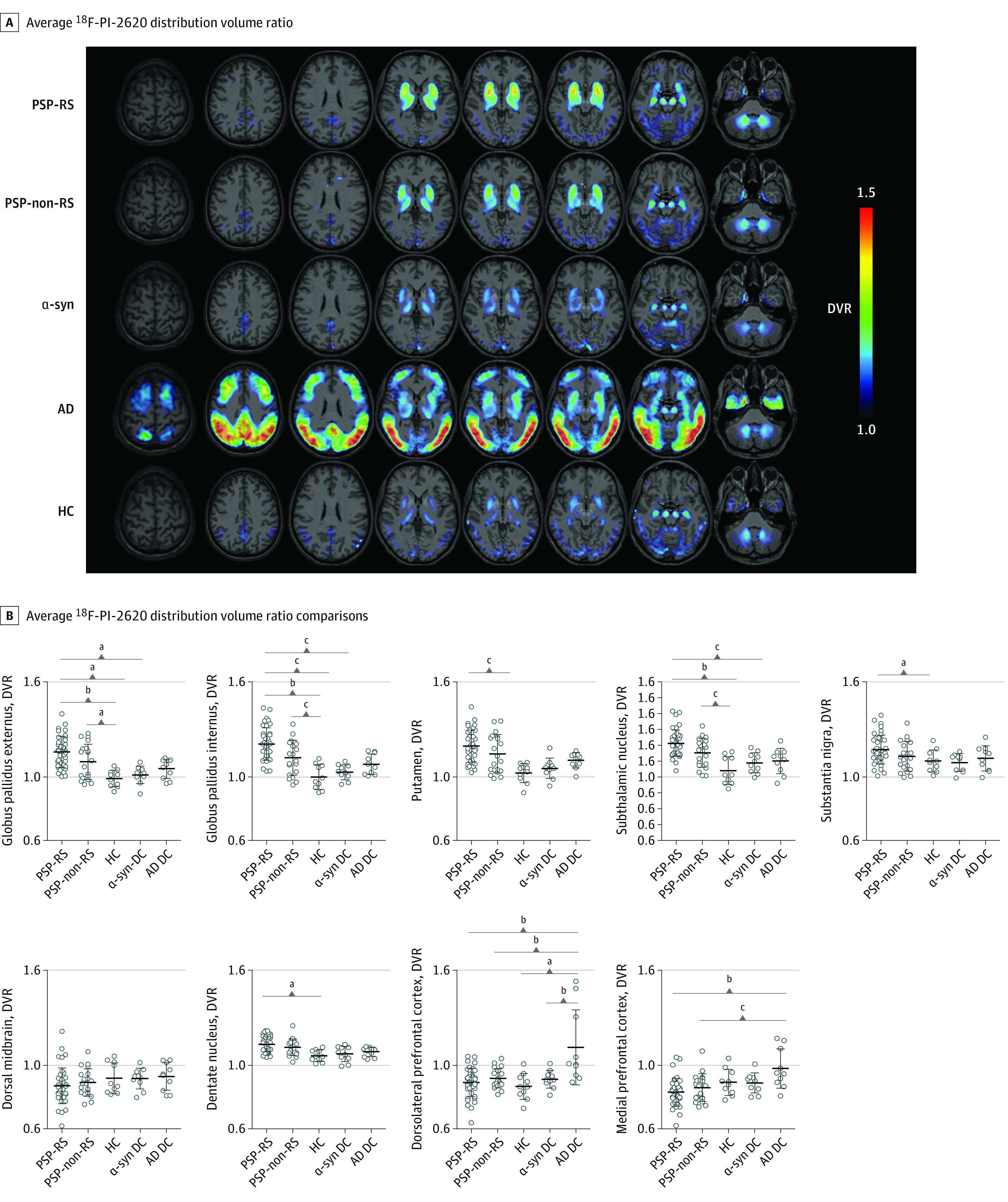
^18^F-PI-2620 Binding in Predefined PSP Target Regions A, Average ^18^F-PI-2620 distribution volume ratio (DVR) binding maps presented as axial overlays on a standard magnetic resonance imaging template for all study groups. Extracerebral voxels were masked. B, ^18^F-PI-2620 DVR comparison between different study groups for the 9 evaluated progressive supranuclear palsy (PSP) target regions. Statistics derive from multivariate analysis of variance including center, age, and sex as covariates and Bonferroni adjustment for multiple comparisons. Error bars indicate mean (SD). α-syn indicates probable α-synucleinopathies; AD, Alzheimer disease; DC, controls with disease; HC, healthy controls; RS, Richardson syndrome. ^a^*P* < .050. ^b^*P* < .001. ^c^*P* < .010.

Significant ^18^F-PI-2620 binding differences among groups were observed for all subcortical PSP target regions except the dorsal midbrain, with strongest differences in the globus pallidus internus. Pairwise group comparisons with post hoc Bonferroni-correction revealed elevated ^18^F-PI-2620 binding in patients with PSP-RS and PSP–non-RS compared with healthy controls and controls with disease in the globus pallidus internus and externus as well as in the subthalamic nucleus (Figure 2 and Table). Patients with PSP-RS also indicated higher binding in the putamen, the substantia nigra, and the dentate nucleus compared with healthy controls, whereas patients with PSP–non-RS did not. The dorsolateral prefrontal cortex and the medial prefrontal cortex indicated lower binding in patients with PSP-RS and PSP–non-RS against patients with AD but no significant binding differences against α-synucleinopathy patients and healthy controls. Exemplary PSP cases with elevated cortical binding are shown in eFigure 4 in the Supplement. For time-activity ratio curves, see eFigure 5 in the Supplement. There was a good to excellent correlation between DVR and 30- to 60-minute SUVr in PSP target regions for the full cohort of 90 individuals (*R^2^*range, 0.45-0.88; eFigure 6 in the Supplement).

**Figure 3.**
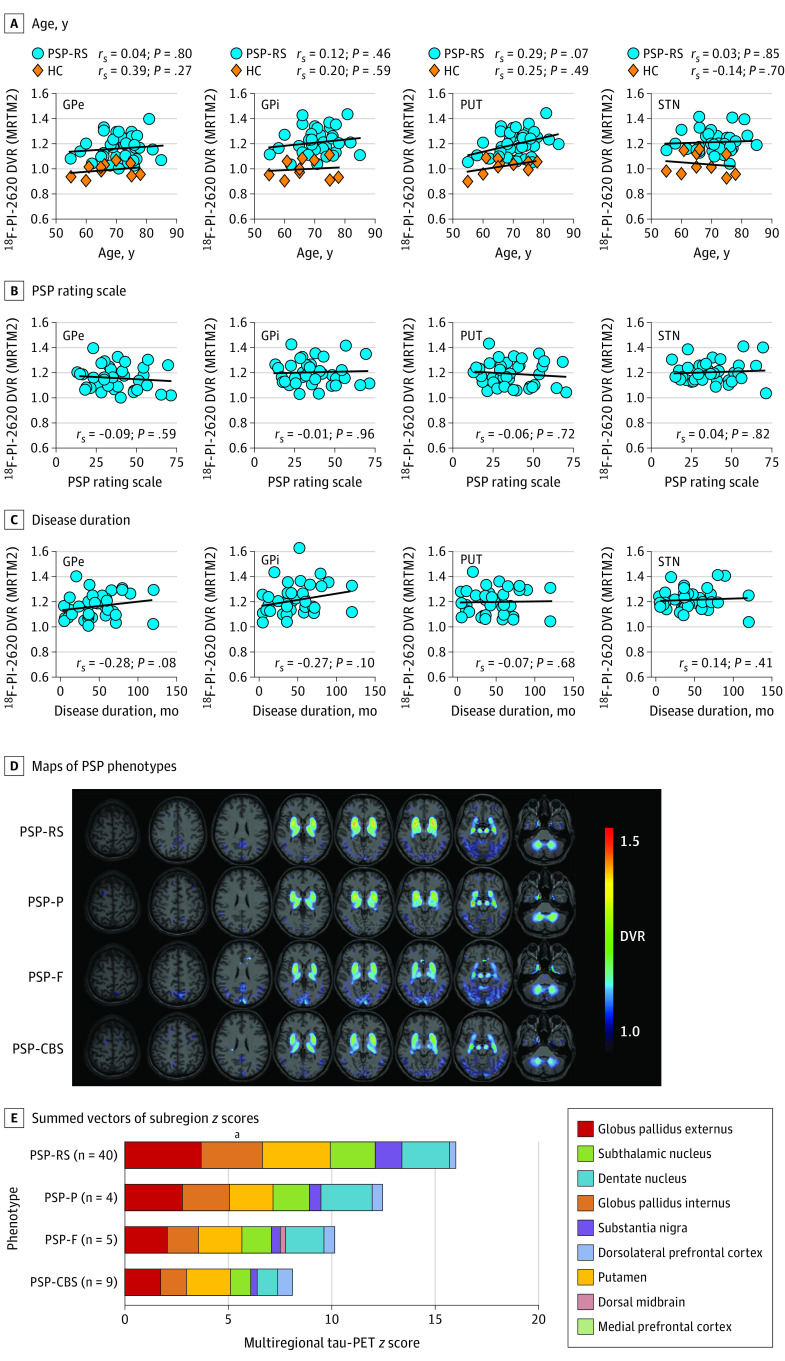
Association of ^18^F-PI-2620 Binding With Age, Disease Severity, Disease Duration, and Phenotype ^18^F-PI-2620 binding as a function of age (A), disease severity (B), and disease duration (C), expressed as correlation plots. Additional plots are in eFigure 9 in the Supplement. *r_S_* indicates Spearman coefficients of correlations. Average ^18^F-PI-2620 distribution volume ratio (DVR) maps of different progressive supranuclear palsy (PSP) phenotypes illustrated by axial slice overlays on a standard magnetic resonance imaging template (D) and quantified by summed vectors of subregion *z* scores (E). GPe indicates globus pallidus externus; GPi, globus pallidus internus; HC, healthy controls; MRTM2, multilinear reference tissue modeling 2; PSP-CBS, PSP with predominant corticobasal syndrome; PSP-F, PSP with predominant frontal presentation; PSP-P, PSP with predominant parkinsonism; PSP-RS, PSP with Richardson syndrome; PUT, putamen; STN, subthalamic nucleus. ^a^Significantly differing regional ^18^F-PI-2620 binding among PSP subtypes.

There was no significant correlation of the ^18^F-PI-2620 DVRs with age for healthy controls or in patients with PSP-RS in PSP target regions with significant elevation in PSP groups, but a significant negative correlation of ^18^F-PI-2620 DVRs with age for healthy controls in the dorsal midbrain and dorsolateral and medial prefrontal cortices (Figure 3A and eFigure 9A in the Supplement). The PSP rating scale values and disease duration were not significantly correlated with ^18^F-PI-2620 binding in patients with PSP-RS (Figure 3B and C and eFigure 9B and C in the Supplement). A subanalysis of the 16 individuals with PSP-RS with low disease severity (PSP rating scale score, ≤30) indicated similar ^18^F-PI-2620 DVR effect sizes vs control groups in comparison with the full PSP-RS cohort, as exemplarily reported for the globus pallidus internus (Cohen *d *for individuals with PSP-RS with low disease severity vs full PSP-RS cohort: healthy controls, 2.3 vs 2.3; Parkinson disease/multiple system atrophy, 2.3 vs 2.2; AD, 1.5 vs 1.5).

Among different phenotypes of PSP, the highest ^18^F-PI-2620 binding was observed in PSP-RS followed by PSP with predominant parkinsonism, PSP with predominant frontal presentation, and PSP with predominant corticobasal syndrome (Figure 3D and E). *z* Scores of ^18^F-PI-2620 binding in the globus pallidus internus indicated significant differences among PSP phenotypes. Subgroup comparisons revealed higher ^18^F-PI-2620 DVR *z* scores in PSP-RS compared with PSP with predominant corticobasal syndrome (mean [SD], 2.68 [1.26] vs 1.45 [1.33]).

### PSP Diagnosis at the Single-Patient Level

**Figure 4.**
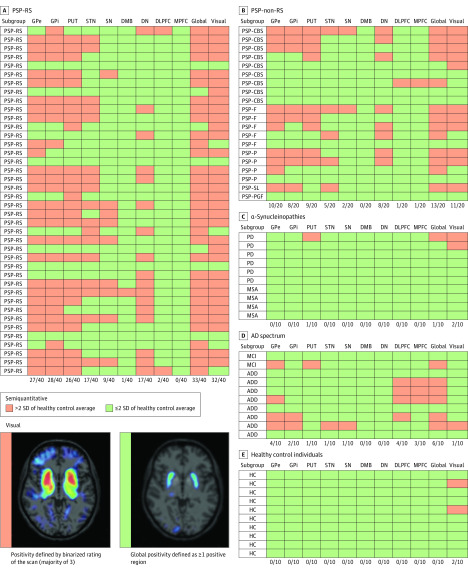
Evaluation of ^18^F-PI-2620 for Detection of Progressive Supranuclear Palsy (PSP) at the Single-Patient Level Semiquantitative classification of PSP target regions. A single region defined the scan as global positive. Visual classification was performed by dichotomous rating of the ^18^F-PI-2620 scan by 3 raters, who defined positivity/negativity for a PSP-like pattern. The bottom of each panel indicates the number of positive regions and total regions. AD indicates Alzheimer disease; ADD, dementia due to AD; DLPFC, dorsolateral prefrontal cortex; DMB, dorsal midbrain; DN, dentate nucleus; GPe, globus pallidus externus; GPi, globus pallidus internus; HC, healthy controls; MCI, mild cognitive impairment due to AD; MPFC, medial prefrontal cortex; MSA, multiple system atrophy; PD, Parkinson disease; PUT, putamen; PSP-CBS, PSP with predominant corticobasal syndrome; PSP-F, PSP with predominant frontal presentation; PSP-P, PSP with predominant parkinsonism; PSP-PGF, PSP with progressive gait freezing; PSP-RS, PSP with Richardson Syndrome; PSP-SL, PSP with predominant speech/language impairment; SN, substantia nigra; STN, subthalamic nucleus.

At the single-patient level, 34 of 40 individuals (85%) with PSP-RS and 12 of 20 individuals (60%) with PSP–non-RS were classified as PET-positive by the semiquantitative approach, yielding a sensitivity of 85% for PSP-RS and 65% for PSP–non-RS (Figure 4). On the other side, only 1 of 10 individuals with Parkinson disease/multiple system atrophy and 6 of 10 controls with AD were classified as PET-positive in PSP target brain regions, while there was no outlier in the healthy control group. This led to an overall specificity of 77% in the combined 30 controls by the semiquantitative approach. The visual read indicated a sensitivity of 80% for PSP-RS and 55% for PSP–non-RS at a specificity of 83% in the combined 30 controls (Figure 4 and eFigures 7 and 8 in the Supplement). Two individuals with Parkinson disease/multiple system atrophy, 1 with AD, and 2 healthy controls were judged positive for a PSP-like pattern. Interreader and test-retest agreements are reported in the eResults in the Supplement.

## Discussion

To our knowledge, we present the first comprehensive in vivo evaluation of a tau-PET tracer with improved off-target binding in patients with clinically diagnosed PSP. In vitro autoradiography experiments on postmortem tissue of 4 individuals with PSP obtained independently from PET imaging showed ^18^F-PI-2620 binding in PSP target regions in colocalization to 4R tau. Our multicenter in vivo data indicate great potential to diagnose patients with suspected PSP using ^18^F-PI-2620 PET. Dichotomous evaluation at the single-patient level yielded high sensitivity and specificity in strong congruence between semiquantitative and visual approaches for the discrimination between patients with clinically diagnosed PSP and controls. Finally, we gained preliminary evidence that the magnitude of ^18^F-PI-2620 binding in PSP target regions differs between different PSP phenotypes, thus pointing to the potential of assessing regional phenotype variability in PSP by in vivo PET imaging (eDiscussion in the Supplement).

The regions with elevated ^18^F-PI-2620 binding in PSP are in line with those found by earlier tau tracers. ^18^F-flortaucipir and ^18^F-THK5351 studies found the strongest binding differences for the globus pallidus, the subthalamic nucleus, and the midbrain when comparing individuals with PSP with healthy controls.^7,8,24,25^ However, large proportions of the basal ganglia signal of ^18^F-THK5351 in individuals with PSP and healthy controls were not specific for tau.^26^ We observed only minor elevation above a DVR of 1.0 in healthy controls for ^18^F-PI-2620 in all brain regions apart from the substantia nigra, suggesting low off-target binding for ^18^F-PI-2620 in PSP target regions. This lower off-target background together with lower variance of tracer binding in PSP target regions of healthy controls for ^18^F-PI-2620 compared with that of earlier tau PET tracers^8,23^ might lead to higher effect sizes in PSP diagnosis. However, proof of this hypothesis would require head-to-head tracer comparison studies. Importantly, we also observed no significant elevations of ^18^F-PI-2620 binding in basal ganglia regions in suspected tau-negative controls with disease (Parkinson disease/multiple system atrophy) compared with healthy controls. Therefore, a relevant ^18^F-PI-2620 off-target binding to α-synuclein or coexisting neuroinflammatory processes seems unlikely. However, we still note that regions with elevated in vivo ^18^F-PI-2620 binding in PSP in our study are known off-target regions of earlier tau ligands. Thus, although the binding in these regions was only low in controls, there could be potential off-target source of a parallel PSP-related pathologic process contributing to the net difference between individuals with PSP and controls. In this regard, it is of interest that no group differences were found in the frontal cortex and the dorsal midbrain in vivo, while in vitro autoradiography revealed a signal in the frontal cortex (eDiscussion in the Supplement). The detailed contributions of possible specific and off-target ^18^F-PI-2620 signal sources in PSP remain to be elucidated by correlative studies between PET and autopsy. Differing topology of neuropathology among individuals, regional differences in target abundance, and an unfavorable regional target-to-atrophy relationship also need to be considered as a potential reason for lacking regional PET differences in vivo.

In contrast to other PSP target regions, there was an elevated DVR (mean, 1.10) for the substantia nigra, which likely is attributable to neuromelanin off-target binding as shown in vitro for tau PET tracers.^13,27^ With regard to disease specificity, we observed a slightly elevated signal in the basal ganglia of some investigated individuals with AD, which has been reported previously.^14^ This finding could be explained by a 2019 Japanese autopsy study, which reported on the presence of tau in the basal ganglia of individuals with AD.^28^ Potential effects of differing ^18^F-PI-2620 binding affinity to 3/4R and 4R tau on the observed basal ganglia signal in AD and on the reported time-activity curves are discussed in the eDiscussion in the Supplement. Identification of an AD-like pattern appeared to be the main advantage of a visual PSP classification, as it facilitated negation of a PSP-like pattern by the reader despite semiquantitative positivity of basal ganglia or frontal cortex target regions. Thus, a combination of both the semiquantitative and visual PET data analysis approaches could increase the specificity for PSP identification when AD is a potential differential diagnosis.

Interestingly, we did not observe a correlation between ^18^F-PI-2620 binding and clinical severity or disease duration in any of the target regions for patients with PSP-RS. Previous correlative results between tau-PET tracer binding and PSP disease severity have been inconsistent, showing no^7^ or positive association.^8,25^ Especially for ^18^F-THK5351, this could be explained by increased monoaminoxidase-B expression in this disorder as a neuroinflammation event. Loss of tracer signal due to partial volume effects caused by increasing atrophy could potentially mask effects in individuals with PSP with long disease duration. Thus, the use of partial volume effect correction on ^18^F-PI-2620 PET data to diagnose PSP will be an interesting task of future studies. Furthermore, there is limited autopsy data investigating tau deposition in PSP as a function of disease duration.^23^ However, longitudinal in vivo imaging data on PSP clearly indicated changes over time in magnetic resonance imaging measures of atrophy but only minor changes of ^18^F-flortaucipir binding.^29^ In summary, large-scale longitudinal studies are needed to investigate the value of ^18^F-PI-2620 as a progression biomarker in PSP.

### Limitations

Among the limitations of our study, the small number of participants needs to be considered. This might mask effects such as age dependency of tracer binding at the current stage. Further, given the nature of an observational multicenter evaluation, we cannot fully rule out effects of disproportional distribution of study groups and phenotype subgroups among centers. Center bias was mitigated by harmonization of the PET data across all sites and inclusion of the center as a covariate in the statistical analyses.

Unpublished analyses by the authors reveal a higher off-rate for ^18^F-PI-2620 from 4R tau when compared with 3/4R tau, which deserves more detailed investigation of binding characteristics. We are aware of the missing autopsy data. Nearly all patients are still alive, and no brains have been donated so far. Thus, we have no pathologic validation of tau positivity for our in vivo results and potential clinical misdiagnoses, especially in the non–RS-PSP cohort, need to be taken into consideration. The current study provides the opportunity of follow-up ^18^F-PI-2620 imaging to study disease progression.

## Conclusions

This multicenter evaluation indicates that ^18^F-PI-2620 PET imaging can aid in diagnosing and differentiating patients with suspected PSP, potentially facilitating a more reliable diagnosis of PSP. Additional studies need to focus on autopsy validation and longitudinal imaging to test if this radiotracer also has potential as a PSP progression biomarker.

## Notes

Supplement. eMethods eResults eDiscussion eReferences eFigure 1. Definition of target and reference regions eFigure 2. Training set for the visual read eFigure 3. Autoradiography visualization and quantification of confirmatory samples eFigure 4. Exemplary PSP patients with cortical 18F-PI-2620 binding eFigure 5. Tracer kinetics in PSP target regions eFigure 6. Agreement between dynamic and static imaging eFigure 7. Axial planes of ^18^F-PI-2620 binding in the basal ganglia of all subjects eFigure 8. Axial planes of ^18^F-PI-2620 binding throughout the brain of all PSP-non-RS subjects eFigure 9. Regional associations of 18F-PI-2620 binding with age, disease severity and disease duration eTable. Extended study overview
