# Supplementary material for: Assessment of 18F-PI-2620 as a Biomarker in Progressive Supranuclear Palsy
Source: JAMA Neurol. 2020 Jul 7;77(11):1–13. doi: 10.1001/jamaneurol.2020.2526 (PMC7341407; doi:10.1001/jamaneurol.2020.2526)
Supplement: Supplement. — eMethods eResults eDiscussion eReferences eFigure 1. Definition of target and reference regions eFigure 2. Training set for the visual read eFigure 3. Autoradiography visualization and quantification of confirmatory samples eFigure 4. Exemplary PSP patients with cortical 18F-PI-2620 binding eFigure 5. Tracer kinetics in PSP target regions eFigure 6. Agreement between dynamic and static imaging eFigure 7. Axial planes of 18F-PI-2620 binding in the basal ganglia of all subjects eFigure 8. Axial planes of 18F-PI-2620 binding throughout the brain of all PSP-non-RS subjects eFigure 9. Regional associations of 18F-PI-2620 binding with age, disease severity and disease duration eTable. Extended study overview [file jamaneurol-e202526-s001.pdf]

Supplementary Online Content

Brendel M, Barthel H, van Eimeren T, et al. Assessment of ^{18}F-PI-2620 as a biomarker in progressive supranuclear palsy. *JAMA Neurol*. Published online July 7, 2020. doi:10.1001/jamaneurol.2020.2526

eMethods

eResults

eDiscussion

eReferences

eFigure 1. Definition of target and reference regions

eFigure 2. Training set for the visual read

eFigure 3. Autoradiography visualization and quantification of confirmatory samples

eFigure 4. Exemplary PSP patients with cortical ^{18}F-PI-2620 binding

eFigure 5. Tracer kinetics in PSP target regions

eFigure 6. Agreement between dynamic and static imaging

eFigure 7. Axial planes of ^{18}F-PI-2620 binding in the basal ganglia of all subjects

eFigure 8. Axial planes of ^{18}F-PI-2620 binding throughout the brain of all PSP-non-RS subjects

eFigure 9. Regional associations of 18F-PI-2620 binding with age, disease severity and disease duration

eTable. Extended study overview

This supplementary material has been provided by the authors to give readers additional information about their work.

© 2020 Brendel et al. *JAMA Neurology.*

eMethods

Radiosynthesis

Radiosynthesis of ^{18}F-PI-2620 was achieved by nucleophilic substitution on a butyloxycarbonyl-protected nitro precursor using an automated synthesis module (IBA, Synthera). The protecting group was cleaved under the radiolabelling conditions. The product was purified by semipreparative high performance liquid chromatography. Radiochemical purity was 99%. Non-decay corrected yields were about 35% with a molar activity of $8 \cdot 10^6$ GBq/mmol at the end of synthesis.

Post mortem brain tissue analyses

Blocks of two regions of Formalin fixed and paraffin-embedded *post mortem* tissue of four Progressive supranuclear palsy (PSP) patients and of four healthy controls were obtained from the NeuroBiobank Munich. All cases were lacking relevant copathology (negative for α-synuclein, β-amyloid, TDP-43, FUS). All samples were prepared equally prior to autoradiography. Two PSP and HC were used for either basal ganglia or frontal cortex evaluation.

Basal ganglia: Both PSP cases used for basal ganglia evaluation had a clinical diagnosis of PSP Richardson syndrome. PSP case #1 was female, died at an age of 67 years, had a *post mortem* interval of 38 hours and a fixation time of 195 days. PSP case #2 was male, died at an age of 70 years, had a *post mortem* interval of 100 hours and a fixation time of 177 days. Healthy control case #1 was female, died at an age of 60 years due to sepsis after pneumonia and had a *post mortem* interval of 20 hours and a fixation time of 47 days. Healthy control case #2 was female, died at an age of 38 years due to pulmonary embolism and had a *post mortem* interval of 27 hours and affixation time of 127 days.

Frontal cortex: Both PSP cases used for frontal cortex evaluation had a clinical diagnosis of PSP Richardson syndrome. PSP case #1 was female, died at an age of 68 years, had a *post mortem* interval of 7 hours and a fixation time of 80 days. PSP case #2 was female, died at an age of 85 years, had a *post mortem* interval of 8 hours and an unknown fixation time. Healthy control case #1 was Male, died at an age of 38 years due to suicide and had a *post mortem* interval of 118 hours and a fixation time of 13 days. Healthy control case #2 was male, died at an age of 26 years due to suicide and had a *post mortem* interval of 73 hours and affixation time of 88 days.

4 µm sections of the basal ganglia and the frontal cortex were mounted on glass slides, deparaffinized incubated with either 2 nM ^{18}F-PI-2620 or 2 nM ^{18}F-PI-2620 plus 10 µM ^{19}F-PI-2620 for one hour (in PBS). Washing was performed by 30% ethanol/PBS for 1 minute, 70% ethanol/PBS for 2 minutes and PBS for 1 minute. After drying at room temperature for 45 minutes (basal ganglia) or 90 minutes (frontal cortex), the sections were placed on Fujifilm BAS cassette2 2025 imaging plates. The plates were exposed for six hours and then scanned at 25 µm resolution with the Raytest equipment (CR-35-BIO, Dürr Medical, Germany). Resulting images were analyzed with a dedicated software (AIDA image analysis, V4.50, Raytest GmbH, Straubenhardt, Germany). Regions of interest were drawn manually in the globus pallidus (internal & external part) and the putamen in a minimum of four slices per sample. The capsula externa served as reference region, and the tracer uptake ratios between target and reference regions were calculated. Hematoxylin and Eosin as well as Kluever-Barrera staining of adjacent slices served for precise anatomical definition of regions of interest and AT8-stained slides were used for colocalization analysis of tracer uptake and tau aggregate load. Regions of interest were manually drawn within the objective slides by use of a conventional microscope. The immunohistochemical tau-staining was performed semi-automatically on a BenchMark device (Ventana, now Hoffmann-LaRoche, Basel, Switzerland) with mouse monoclonal AT8 antibody raised against hyperphosphorylated tau (Ser202/Thr205; Innogenetics, Gent, Netherlands).

Acquisition, reconstruction and image harmonization

^{18}F-PI-2620 positron-emission-tomography (PET) imaging in combination with computed tomography (CT) or magnetic resonance (MR) was performed in a full dynamic setting (minimum scan duration: 0 – 60 min p.i.) on different scanners at five specialized neuroimaging sites using the established standard parameter of each center for brain PET imaging as follows:

<u>Munich:</u> The PET data in Munich were acquired on a Siemens Biograph True point 64 PET/CT (Siemens, Erlangen, Germany) or a Siemens mCT (Siemens, Erlangen, Germany). The dynamic brain PET data were acquired in 3-dimensional list-mode over 60min, and reconstructed into a 336x336x109 matrix (voxel size: $1.02\times1.02\times2.03$ mm^3) using the built-in ordered subset expectation maximization (OSEM) algorithm with 4 iterations, 21 subsets and a 5mm Gaussian filter. A low dose CT served for attenuation correction.

<u>Leipzig:</u> The PET data in Leipzig were acquired on a hybrid PET/MR system (Biograph mMR, Siemens Healthineers, Erlangen, Germany). The dynamic brain PET data were acquired in 3D list-mode over 60 min, and reconstructed into a 256x256 matrix (voxel size: $1.00\times1.00\times2.03$ mm^3) using the built-in ordered subset expectation maximization algorithm with 8 iterations, 21 subsets and a 3mm Gaussian filter. For attenuation correction, the vendor-provided HiRes method was employed. This method combines the individual Dixon

© 2020 Brendel et al. *JAMA Neurology.*

attenuation correction approach with a bone attenuation template[1]. As such, it is considered accurate[1], guaranteeing the use of the respective site PET data in this multi-center study.
Cologne: The PET data in Cologne were acquired on a Siemens mCT (Siemens, Erlangen, Germany). The dynamic brain PET data were acquired in 3D list-mode over 60min, and reconstructed into a 400x400 matrix (effective voxel size: $1.02\times1.02\times1.50$ mm^3) using the built-in ordered subset expectation maximization algorithm with 4 iterations, 12 subsets and a 5 mm Gaussian filter. A low dose CT served for attenuation correction.
New Haven: Subjects were positioned in a gently-securing head holder using the laser lights of the camera so that the brain was centered in the field of view. Prior to the radiotracer injection and emission imaging, a transmission scan was performed with an external ^{68}Ge rod source to provide correction coefficients for photon attenuation in the matter. Subjects were administered a single dose of ^{18}F-PI-2620 through a venous catheter followed by a 10 mL saline flush. Dynamic PET imaging of the brain was acquired on a Siemens ECAT EXACT HR+ camera from 0-90 and 120-180 min. Images were reconstructed in a 128 x 128 matrix (zoom=2, pixel size of 2.574 mm x 2.574 mm) with an iterative reconstruction algorithm (OSEM 4 iterations, 16 subsets) and a post hoc Gaussian filter = 5 mm. Standard corrections for random, scatter, system dead time and attenuation provided by the camera manufacturer were performed.
Melbourne: Acquisition was performed by a Philips Gemini TF 64 PET/CT (Philips, Eindhoven, The Netherlands). Brain PET images were acquired dynamically from 0-60 min and 80-120 min (5-minute frames) minutes post injection. Images were reconstructed using LOR-RAMLA and CT attenuation correction was performed. Images were binned into a 128x128x89 matrix (voxel size: $2.00\times2.00\times2.00$ mm^3).
Frame binning was standardized to 6 x 30 seconds, 4 x 60 seconds, 4 x 120 seconds, and 9 x 300 seconds.
The injected dose was 168 to 334 MBq and applied as a bolus injection. Data from Hofmann brain phantoms were used to obtain scanner-specific filter functions which were then consequently used to generate images with a similar spatial resolution (full-width-at-half-maximum=9x9x10mm; determined by the scanner in New Haven), following the Alzheimer's Disease Neuroimaging Initiative image harmonization procedure[2]. Resulting smoothing factors were 3.5x3.5x7.0mm for Munich, 6.0x6.0x6.0mm for Leipzig, 7.0x7.0x7.0mm for Cologne and 4.0x4.0x4.0mm for Melbourne. All dynamic datasets were visually checked and automatically corrected for head motion when necessary using PMOD (V3.9 PMOD technologies Basel, Switzerland).

PET image post-processing
An ^{18}F-PI-2620 PET template was generated to allow inclusion of patients not eligible to an MRI (because of pacemakers, metal implants etc.). 20 randomly chosen datasets of PSP patients (five PSP-RS and two PSP-non-RS from Munich, three PSP-RS and one PSP-non-RS from Leipzig, two PSP-RS from New Haven), disease controls (two AD from Munich and one AD from New Haven, one PD and one MSA from Munich) and healthy controls (two from New Haven) who all had a T1w 3D MRI sequence were automatically processed by the PNEURO pipeline[3] to obtain ^{18}F-PI-2620 images in the Montreal Neurology Institute (MNI) space. Frames between 30 and 60 minutes p.i. were summed and scaled by the global mean, followed by averaging all 20 cases to an ^{18}F-PI-2620 template in the MNI space.
All 90 dynamic ^{18}F-PI-2620 PET datasets were transformed to the MNI space using the non-linear brain normalization of the summed 30-60 min frames to the established ^{18}F-PI-2620 template. Automatized brain normalization settings in PMOD included nonlinear warping, 8 mm input smoothing, equal modality, 16 iterations, frequency cutoff 3, regularization 1.0, and no thresholding. The transformation was saved and applied to the full dynamic ^{18}F-PI-2620 PET datasets to minimize interpolation.

Visual read
Three expert readers independently assessed the ^{18}F-PI-2620 DVR maps in 3D mode using standardized settings (lower/upper DVR threshold 1.0/1.5; spectrum color, overlay on a MRI standard template in the MNI space with 50% transparency) as presented by the Papaya viewer online platform and blinded to the identity of the subject. Five PSP (three PSP-RS and two PSP-non-RS; all scanned in Munich after data lock in October 2019) and five different control cases (three scanned in Munich after data lock in October 2019 and two scanned in New Haven with not matching age) were used as a training-set (see **eFigure 2**). 20% randomly chosen cases of each diagnosis category were repeatedly presented to the readers to assess test-retest variability. Each reader had to score the overall pattern of the scan as positive or negative for a PSP-typical ^{18}F-PI-2620 binding. In particular, the reader was instructed to evaluate binding in putamen, globus pallidus, subthalamic nucleus, substantia nigra and the dentate nucleus and the final decision of positivity or negativity of a PSP-typical pattern was dichotomous for the entire scan. Readers were allowed to take AD-like cortical binding into consideration. The majority judgment from the three readers defined positivity and negativity for a PSP-like ^{18}F-PI-2620 PET scan. Fleiss κ was determined as a measure of intra-reader agreement, and Cohens κ as a measure of agreement between semiquantitative and visual analyses, and for test-retest evaluation.

© 2020 Brendel et al. *JAMA Neurology.*

eResults

Tracer kinetics

Time-activity-ratio-curves between 0-60 minutes showed inverted U-shapes for both parts of the globus pallidus, the putamen, and the subthalamic nucleus, whereas a plateau or a slightly increasing shape was observed for the substantia nigra, the dorsal midbrain and the dentate nucleus. The frontal cortex target regions of Alzheimer's disease patients indicated increasing time-activity-ratios.

Visual read

The inter-reader agreement was moderate to substantial (Fleiss κ=.594, p<.001). The agreement between semiquantitative and visual classification was substantial (Cohens κ=.642, p<.001). Test-retest agreement was excellent (Cohens κ=.810, p<.001), with congruent negative reading in 20 (95%) scans and congruent positive reading in 29 (88%) scans. Representative basal ganglia slices of all subjects and slices throughout the brain of all PSP-non-RS subjects are provided in **eFigures 7&8**

© 2020 Brendel et al. *JAMA Neurology.*

eDiscussion

PSP Phenotype

Our analysis entails a preliminary comparison of ^{18}F-PI-2620 binding in different PSP phenotypes. Keeping limited sample sizes among the more rare PSP subtypes in mind, we observed a roughly matching magnitude of tau-PET signal in different PSP phenotypes when compared with their amount of tau found in autopsy[4]. Furthermore, we found a positive ^{18}F-PI-2620 signal in more than half of the PSP-non-RS patients, potentially allowing early identification of underlying PSP pathology in these underdiagnosed cases[5].

Frontal cortex

Findings related to ^{18}F-PI-2620 binding in the frontal cortex of PSP patients indicated differences among the different applied analyses *in vivo* and *in vitro*. *In vitro* autoradiography showed a clear and co-localized cortical ^{18}F-PI-2620 binding in two cases with high AT8-positive tau load in the frontal cortex. In contrast, there was no elevated ^{18}F-PI-2620 signal in the *in vivo* PET data in the PSP groups and healthy controls at the group level when using predefined frontal cortical regions of interest. By visual inspection of the PET data of single patients, we observed a clearly defined frontal cortex signal only in some PSP cases (see **eFigure 4**), but no relevant frontal cortex ^{18}F-PI-2620 binding in the majority of the investigated PSP patients. This was also reflected by the results of the semi-quantitative analysis, indicating elevations of DVR above the mv ± SD threshold in the dorsolateral and the medial prefrontal cortex in only three PSP cases. Interestingly, the PSP-F patients studied did not show a higher PET signal in frontal cortical target regions when compared to other PSP phenotypes (see **eFigure 8**). Nonetheless, it needs to be considered that the cortical tau load of the studied *in vivo* cases was likely below the tau load of (usually more advanced) autopsy cases. Thus, we speculate that the abundance of tau in this brain area might be too low for the *in vivo* detection threshold of the tracer. Partial volume effects due to pronounced atrophy of frontal cortical regions in PSP patients could be another explanation of the missing group differences. Negative correlations of the ^{18}F-PI-2620 signal with age in the dorsolateral and the medial prefrontal cortex in the limited sample of healthy controls may support this hypothesis as partial volume effects due to age-related volume loss would fit to the observed associations. Future studies need to address if individual standardized assessments (i.e. by 3-dimensional Z-score maps) or partial volume effect correction of frontal cortical ^{18}F-PI-2620 binding have a value for tau PET imaging in PSP.

Binding characteristics

Binding characteristics in subcortical PSP target regions appeared different when compared to previously reported cortical Alzheimer's disease (AD) binding[6]. Due to different ^{18}F-PI-2620 binding characteristics to AD (--$\log_{10}$ of the half maximal inhibitory concentration: 8.5±0.1) and PSP (--$\log_{10}$ of the half maximal inhibitory concentration: 7.7±0.1) brain tissue[7], it might be the case that even low deposition of 3/4R tau in the basal ganglia of AD could cause a higher PET signal as strong 4R tau deposition in the basal ganglia of PSP cases. Except for the substantia nigra and the dorsal midbrain we observed decreasing time-activity ratio curves until 60 minutes scan time after a peak within the first 30 minutes. The dentate nucleus even showed differences against healthy controls only in the first 30 minutes but revealed converging binding ratios in the later acquisition phase. In contrast, cortical AD time-activity ratio curves were reported stable or even increasing with time until 180 minutes[6]. A potential factor explaining the different uptake dynamics could be the already discussed lower affinity of ^{18}F-PI-2620 to 4-repeat (4R) PSP tau when compared to 3/4R AD tau. Consequently, there might be more dissolution of ^{18}F-PI-2620 from 4R tau in contrast to 3/4R tau. Taking the fast wash out of the tracer into consideration this could explain target-to-reference-tissue uptake differences in the late PET image acquisition phase. Second, the subcortical localization of PSP target regions in contrast to cortical AD target regions could also contribute to differences in tracer delivery. A direct comparison of cortical ^{18}F-PI-2620 tau binding between 4R and 3/4R tauopathies might allow elucidating this phenomenon, and the contrast between β-amyloid positive and β-amyloid negative patients with corticobasal syndrome could serve for this purpose[8]. Differences in affinity of ^{18}F-PI-2620 to different tau isoform is also supported by our autoradiography findings, showing a discernible but lower signal in the basal ganglia of PSP tissue when compared to earlier reported autoradiography signal in cortical AD tissue[7].

© 2020 Brendel et al. *JAMA Neurology.*

eReferences

1. Ladefoged CN, Law I, Anazodo U, et al. A multi-centre evaluation of eleven clinically feasible brain PET/MRI attenuation correction techniques using a large cohort of patients. *NeuroImage.* 2017;147:346-359.
2. Jagust WJ, Landau SM, Koeppe RA, et al. The Alzheimer's Disease Neuroimaging Initiative 2 PET Core: 2015. *Alzheimers Dement.* 2015;11(7):757-771.
3. Brendel M, Wagner L, Levin J, et al. Perfusion-Phase [(18)F]THK5351 Tau-PET Imaging as a Surrogate Marker for Neurodegeneration. *J Alzheimers Dis Rep.* 2017;1(1):109-113.
4. Boxer AL, Yu JT, Golbe LI, Litvan I, Lang AE, Hoglinger GU. Advances in progressive supranuclear palsy: new diagnostic criteria, biomarkers, and therapeutic approaches. *The Lancet Neurology.* 2017;16(7):552-563.
5. Jabbari E, Holland N, Chelban V, et al. Diagnosis Across the Spectrum of Progressive Supranuclear Palsy and Corticobasal Syndrome. *JAMA Neurol.* 2019.
6. Mueller A, Bullich S, Barret O, et al. Tau PET imaging with (18)F-PI-2620 in patients with Alzheimer's disease and healthy controls: a first-in-human study. *J Nucl Med.* 2019.
7. Kroth H, Oden F, Molette J, et al. Discovery and preclinical characterization of [(18)F]PI-2620, a next-generation tau PET tracer for the assessment of tau pathology in Alzheimer's disease and other tauopathies. *Eur J Nucl Med Mol Imaging.* 2019;46(10):2178-2189.
8. Rosler TW, Tayaranian Marvian A, Brendel M, et al. Four-repeat tauopathies. *Prog Neurobiol.* 2019;180:101644.
9. Keuken MC, Bazin PL, Backhouse K, et al. Effects of aging on T(1), T(2)*, and QSM MRI values in the subcortex. *Brain Struct Funct.* 2017;222(6):2487-2505.
10. Fan L, Li H, Zhuo J, et al. The Human Brainnetome Atlas: A New Brain Atlas Based on Connectional Architecture. *Cereb Cortex.* 2016;26(8):3508-3526.
11. Carlen M. What constitutes the prefrontal cortex? *Science.* 2017;358(6362):478-482.
12. Hammers A, Allom R, Koepp MJ, et al. Three-dimensional maximum probability atlas of the human brain, with particular reference to the temporal lobe. *Hum Brain Mapp.* 2003;19(4):224-247.

© 2020 Brendel et al. *JAMA Neurology.*

eFigure. Definition of target and reference regions

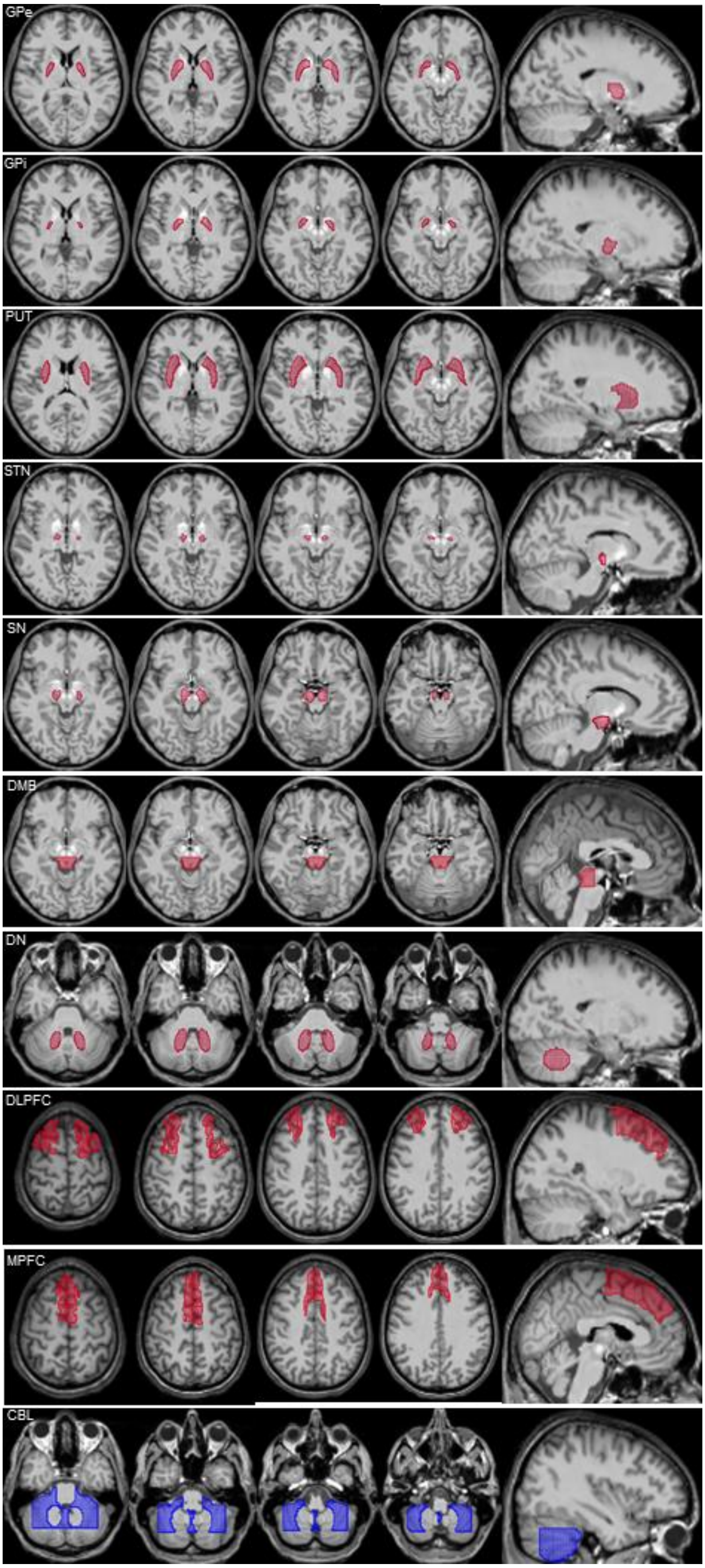

© 2020 Brendel et al. *JAMA Neurology.*

Definition of target (red) and reference (blue) regions in the Montreal Neurology Institute (MNI) space. Outlined volumes-of-interest are projected upon an MRI template. The Atlas of the basal ganglia[9] was used for definition of most subcortical volumes-of-interest (globus pallidus externus and internus, putamen, subthalamic nucleus, substantia nigra). The dorsolateral prefrontal cortex and the medial prefrontal cortex were defined by the Brainnetome atlas[10,11]. The cerebellum, the dentate nuclues and the dorsal midbrain were defined by the Hammers atlas[12]. For delineation of subcortical volumes-of-interest by the Atlas of the basal ganglia, a filter of 9x9x10 mm and a border threshold of 0.1 were applied. The standard grey matter threshold of 0.3 in PMOD was set for definition of voxels in the dorsolateral and medial prefrontal cortices by the Hammers atlas. Superior and posterior layers (1.5 cm) of the cerebellum were excluded manually in the MNI space. The dentate nucleus and the central cerebellar white matter were also excluded. Ventral parts of the midbrain and the lower parts of the brainstem were excluded for definition of the dorsal midbrain. GPe = globus pallidus externus; GPi = globus pallidus internus; PUT = putamen; STN = subthalamic nucleus; SN = substantia nigra; DMB = dorsal midbrain; DN = dentate nucleus; DLPFC = dorsolateral prefrontal cortex; MPFC = medial prefrontal cortex; CBL = cerebellum

© 2020 Brendel et al. *JAMA Neurology.*

eFigure 2. Training set for the visual read

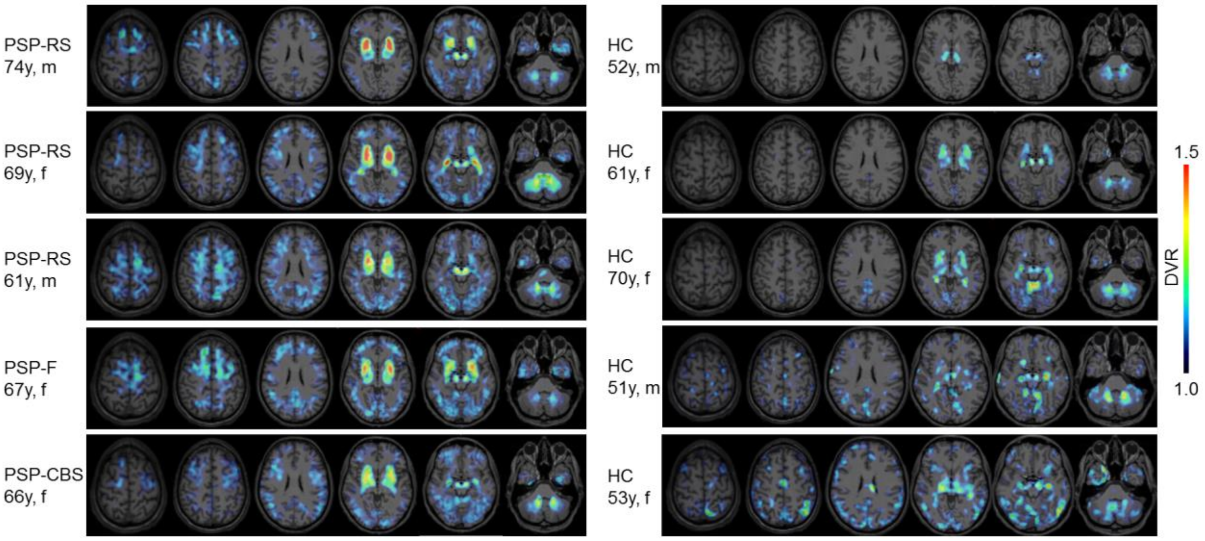

^{18}F-PI-2620 binding maps throughout the brain of all training set subjects presented as axial overlays upon a magnetic resonance standard template. Extra-cerebral voxels were masked. Age in years (y) and sex are provided for each subject but were not available to the reader. DVR = distribution volume ratio; PSP-RS = PSP Richardson syndrome; PSP-CBS = PSP with predominant corticobasal syndrome; PSP-F = PSP with predominant frontal presentation; f = female; m = male

© 2020 Brendel et al. *JAMA Neurology.*

eFigure 3. Autoradiography visualization and quantification of confirmatory samples

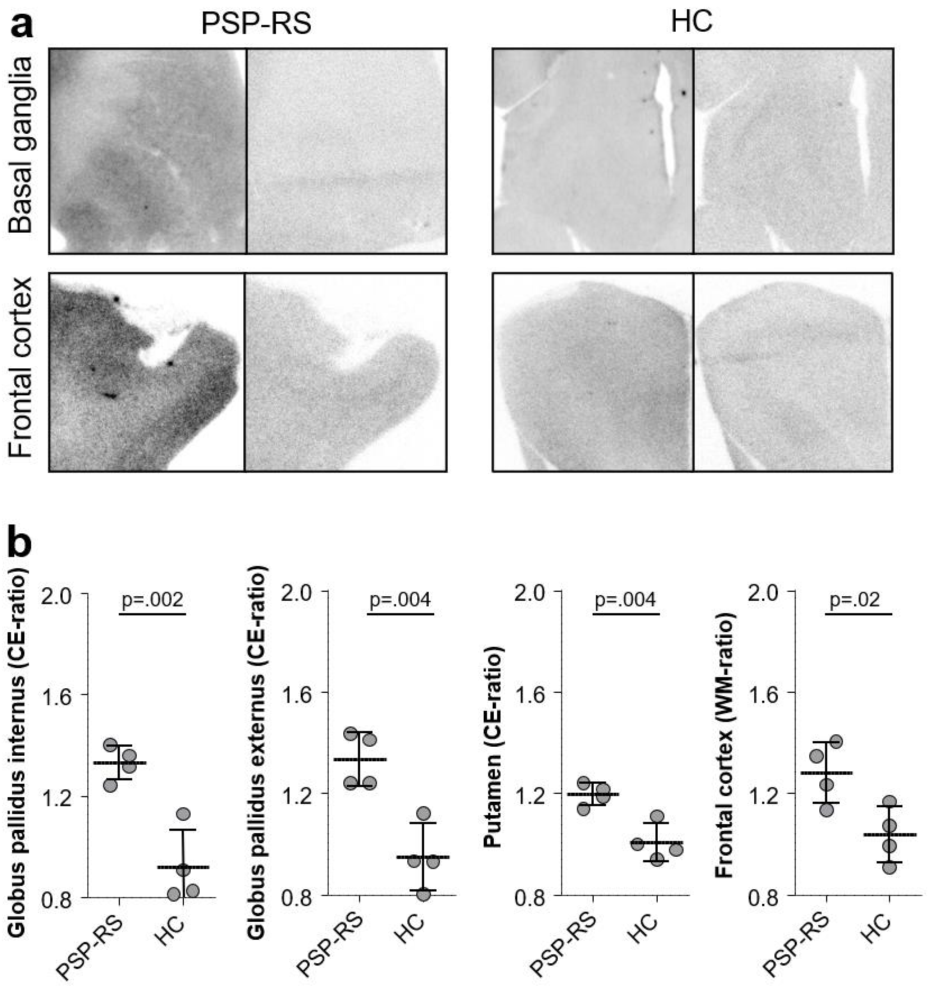

(**a**) Upper row: Autoradiograms of basal ganglia slices of a 70-year old male with a *post mortem* diagnosis of PSP-RS after incubation with ^{18}F-PI-2620 or ^{18}F-PI-2620 and excessive non-labeled compound (^{19}F-PI-2620) as well as autoradiograms of basal ganglia slices of a 38-year old female healthy control. Lower row: Autoradiograms of frontal cortex slices of a 85-year old female with a *post mortem* diagnosis of PSP-RS after incubation with ^{18}F-PI-2620 or ^{18}F-PI-2620 and excessive non-labeled compound (^{19}F-PI-2620) as well as autoradiograms of frontal cortex slices of a 26-year old male healthy control. (**b**) Quantification of ARG binding by region of interest analysis (basal ganglia: target-to-capsula-externa (CE)-ratios; frontal cortex: target-to-white matter (WM)-ratios). Four brain slices of each PSP-RS and healthy control sample were analyzed and the resulting data were compared by a Student's *t*-test. Patient details are provided in the **eMethods** above. PSP = Progressive supranuclear palsy; RS = Richardson syndrome; HC = healthy controls

© 2020 Brendel et al. *JAMA Neurology.*

eFigure 4. Exemplary PSP patients with cortical ^{18}F-PI-2620 binding

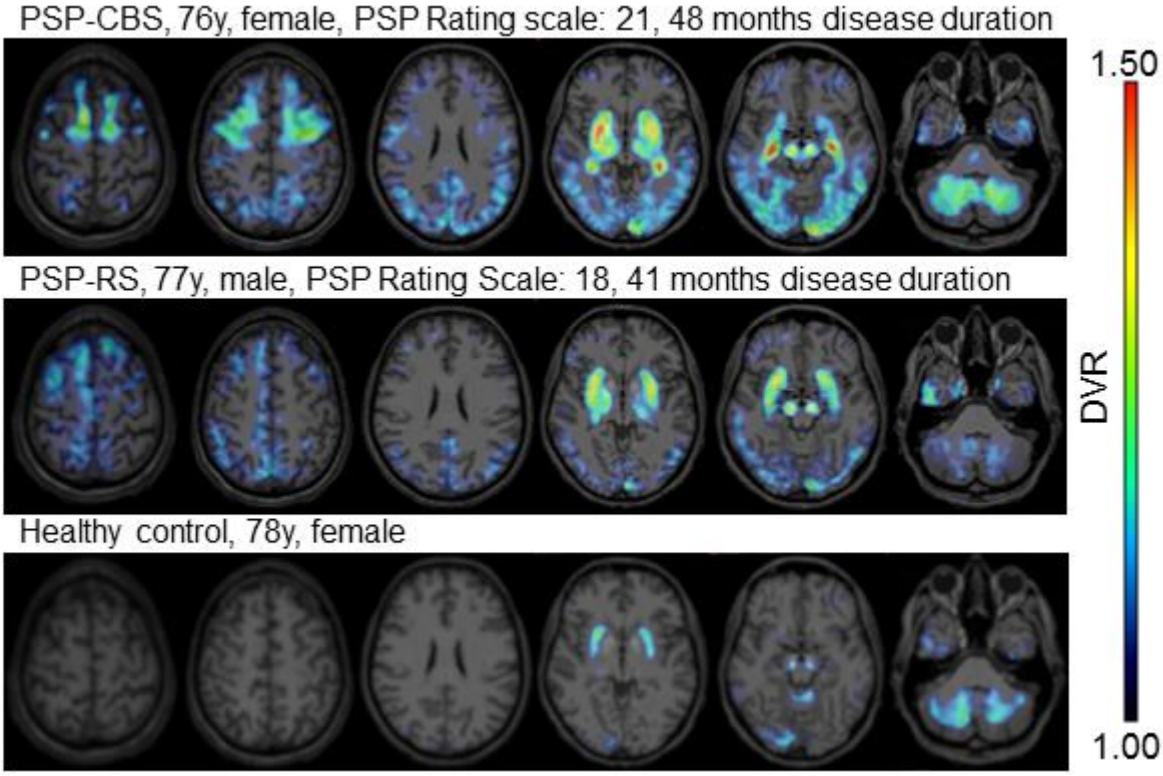

Exemplary patients with elevated cortical ^{18}F-PI-2620 binding in contrast to an age-matched healthy control. Distribution volume ratio (DVR) maps are presented as axial overlays upon a magnetic resonance standard template. PSP-CBS = PSP with predominant corticobasal syndrome; PSP-RS = PSP Richardson syndrome; y = years

© 2020 Brendel et al. *JAMA Neurology.*

eFigure 5. Tracer kinetics in PSP target regions

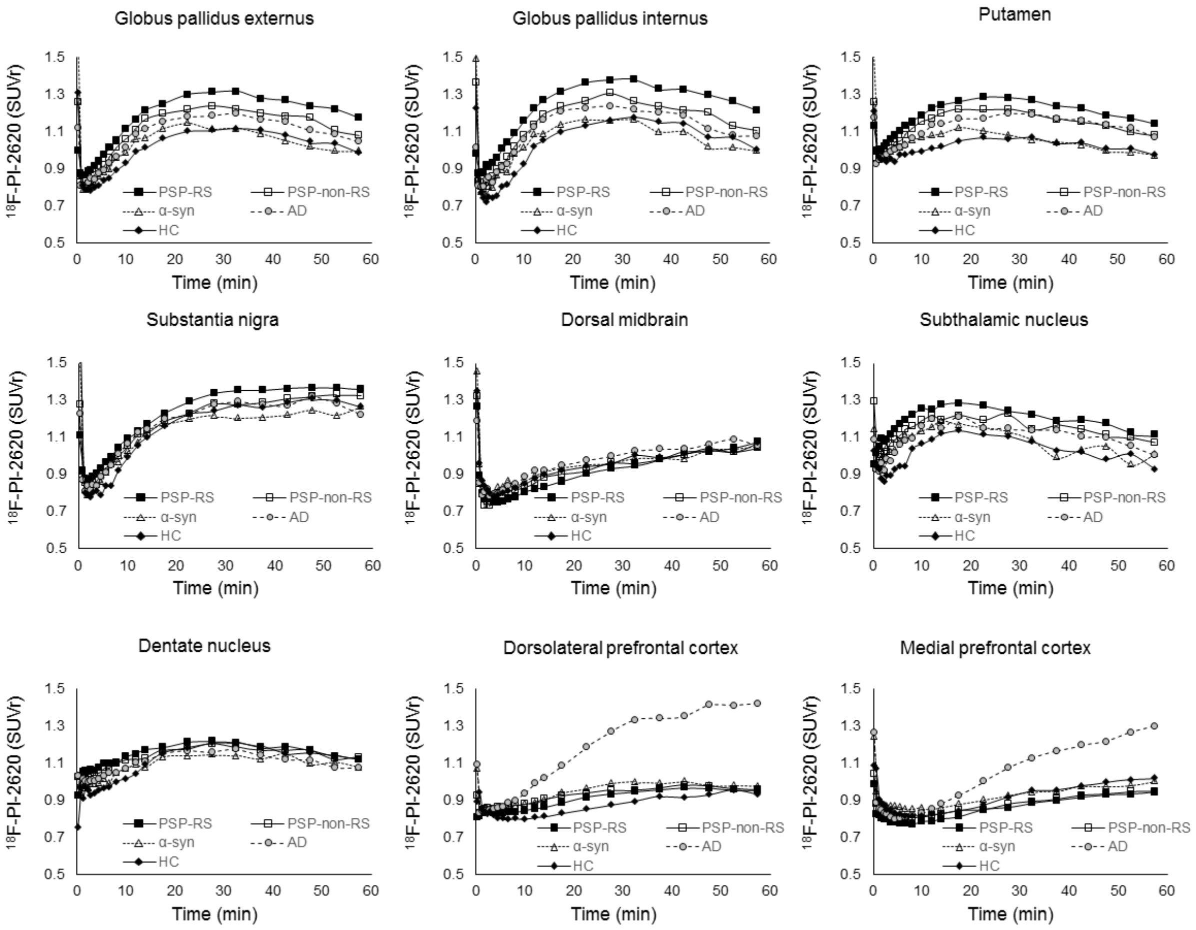

Time-SUVr-curves of all target regions. PSP = Progressive supranuclear palsy; RS = Richardson syndrome; AD = Alzheimer's disease; HC = healthy controls; α-syn = probable α-synucleinopathies; SUVr = standard-uptake-value-ratio; min = minutes

© 2020 Brendel et al. *JAMA Neurology.*

eFigure 6. Agreement between dynamic and static imaging

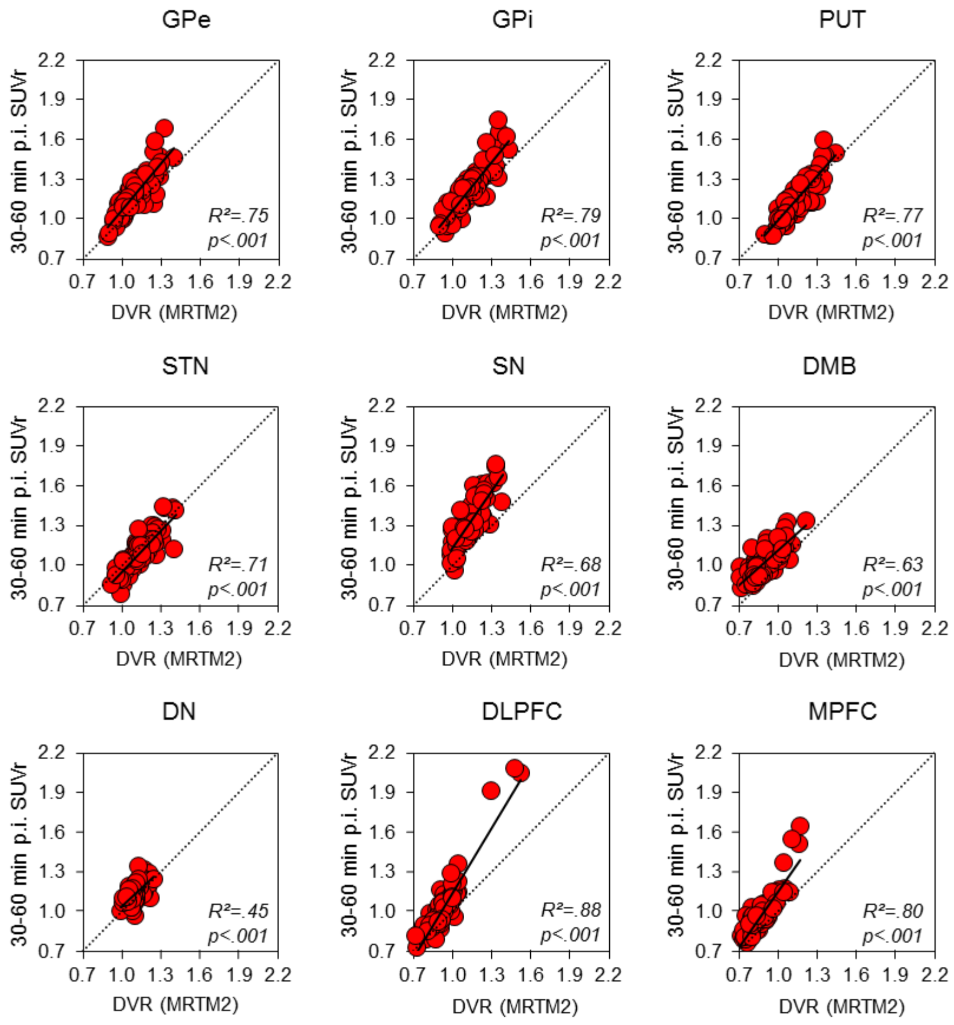

Agreement between distribution volume ratios (DVR) and standardized-uptake-value-ratios (SUVr) from 30 to 60 minute post injection. GPe = globus pallidus externus; GPi = globus pallidus internus; PUT = putamen; STN = subthalamic nucleus; SN = substantia nigra; DMB = dorsal midbrain; DN = dentate nucleus; DLPFC = dorsolateral prefrontal cortex; MPFC = medial prefrontal cortex; MRTM2 = multilinear reference tissue modelling 2. R^2 derive from the correlation of the full dataset of 90 subjects

© 2020 Brendel et al. *JAMA Neurology.*

eFigure 7. Axial planes of ^{18}F-PI-2620 binding in the basal ganglia of all subjects

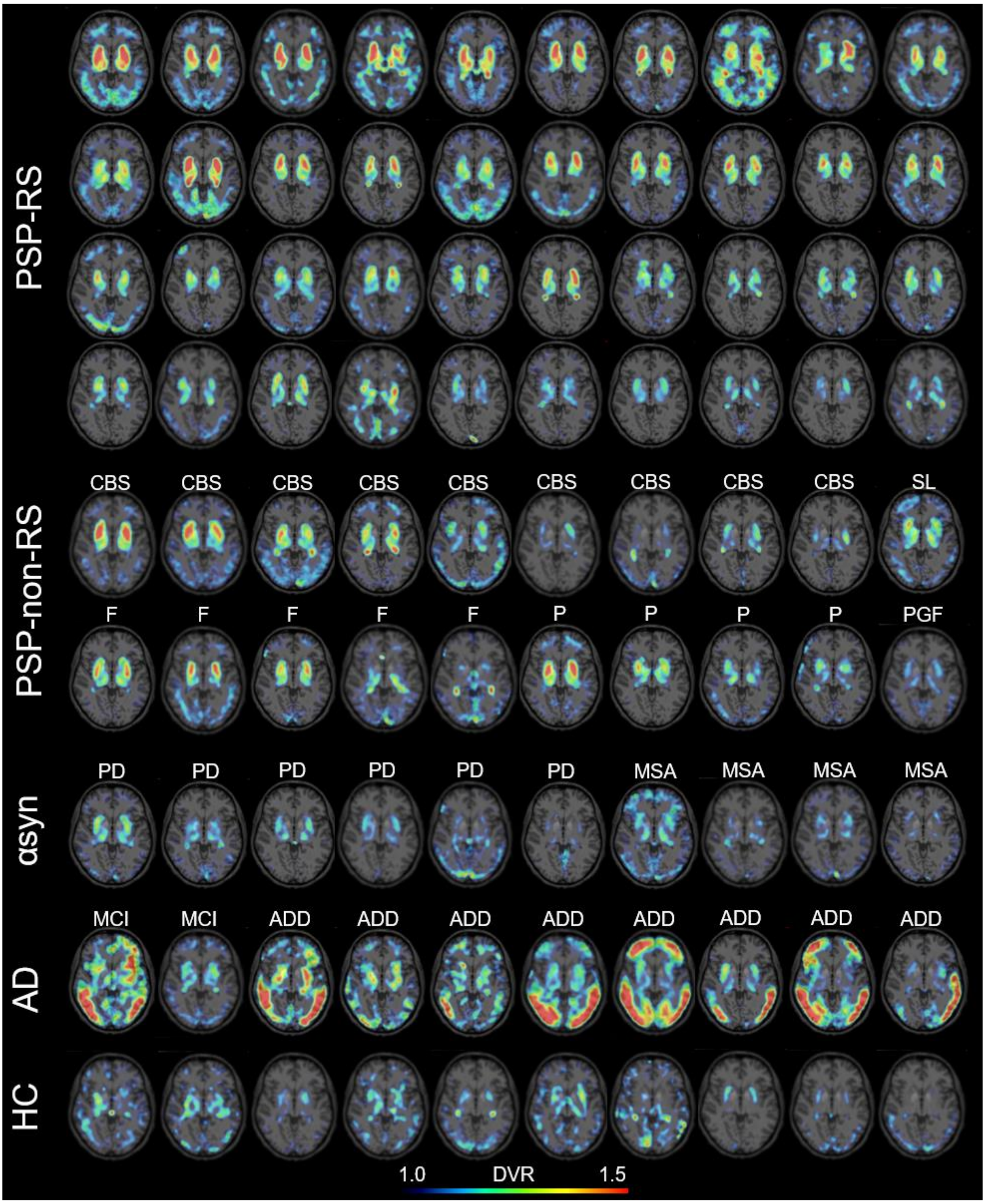

^{18}F-PI-2620 binding maps of the basal ganglia of all 90 subjects presented as axial overlays upon an MRI template. Each axial map represents a single subject. Images of subgroups were sorted by the visual impression of basal ganglia binding. Extra-cerebral voxels were masked. Subgroups are indicated above the image when appropriate. PSP = Progressive supranuclear palsy; RS = Richardson syndrome; AD = Alzheimer's disease; HC = healthy controls; a-syn = suspected α-synucleinopathies; DVR = distribution volume ratio; CBS = PSP with predominant corticobasal syndrome; SL = PSP with predominant speech/language impairment; F = PSP with predominant frontal presentation; P = PSP with predominant Parkinsonism; PGF = PSP with progressive gait freezing; PD = Parkinson's disease; MSA = multiple system atrophy; MCI = mild cognitive impairment due to AD; ADD = dementia due to AD

© 2020 Brendel et al. *JAMA Neurology.*

eFigure 8. Axial planes of ^{18}F-PI-2620 binding throughout the brain of all PSP-non-RS subjects

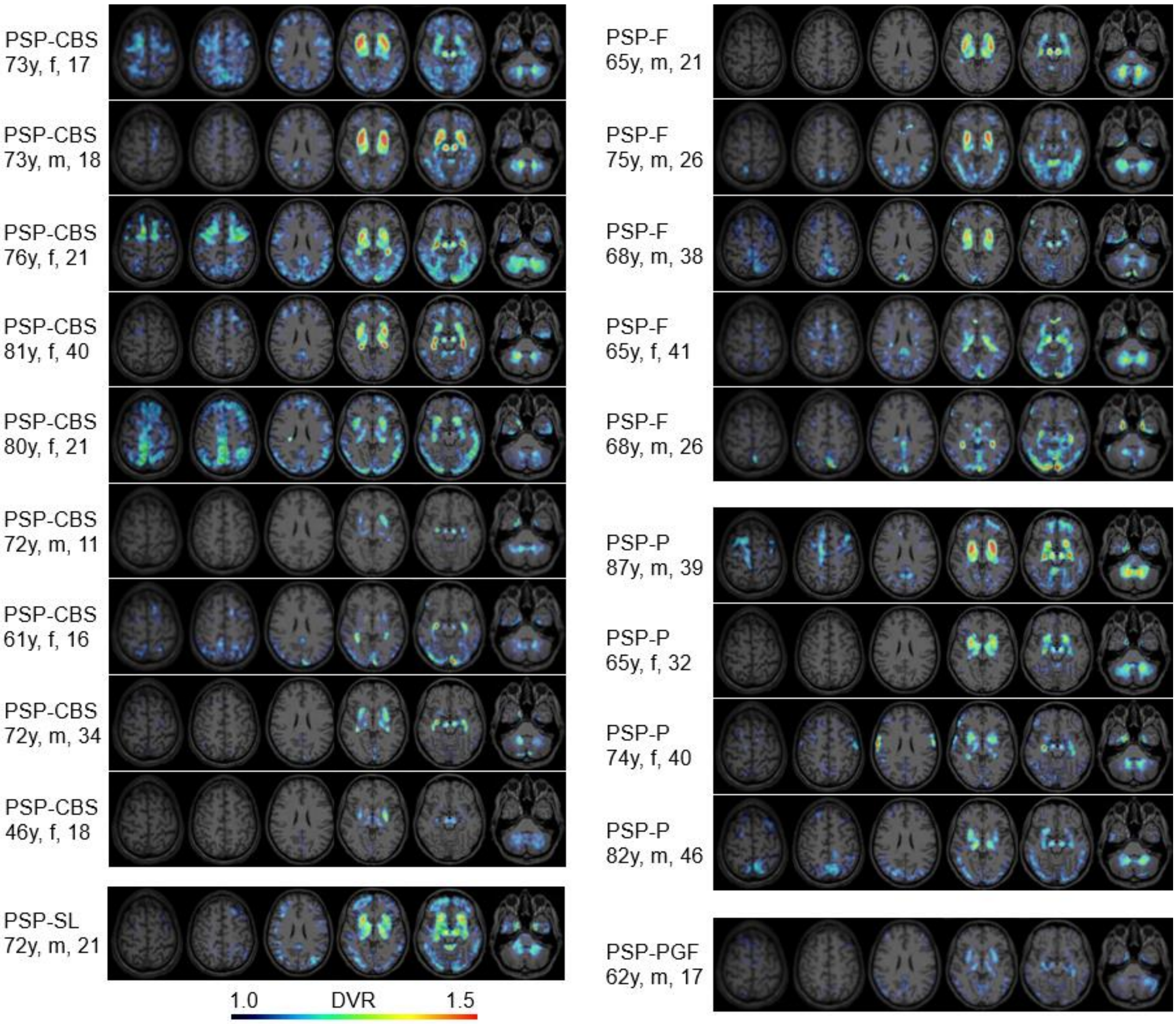

^{18}F-PI-2620 binding maps throughout the brain of all 20 subjects with a non-Richardson syndrome phenotype of Progressive supranuclear palsy (PSP) presented as axial overlays upon a magnetic resonance standard template. Extra-cerebral voxels were masked. Age in years (y), gender, and the PSP Rating Scale score are provided for each subject. DVR = distribution volume ratio; PSP-CBS = PSP with predominant corticobasal syndrome; PSP-SL = PSP with predominant speech/language impairment; PSP-F = PSP with predominant frontal presentation; PSP-P = PSP with predominant Parkinsonism; PSP-PGF = PSP with progressive gait freezing; f = female; m = male

© 2020 Brendel et al. *JAMA Neurology.*

eFigure 9. Regional associations of 18F-PI-2620 binding with age, disease severity and disease duration

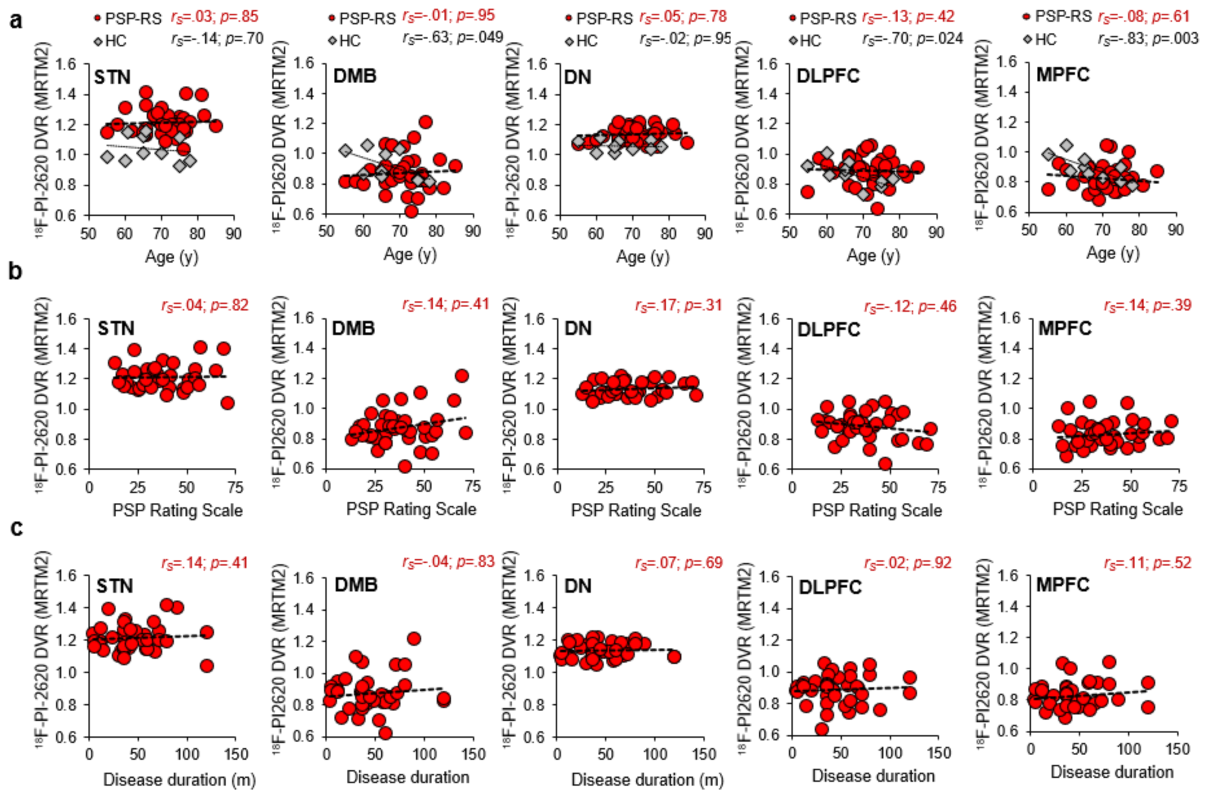

^{18}F-PI-2620 binding as a function of age (**a**), disease severity (**b**), and disease duration (**c**), expressed as correlation plots. r_S indicate Spearman's coefficients of correlations. PSP-RS = red; HC = grey. PSP-RS = Progressive supranuclear palsy Richardson syndrome; HC = healthy controls; DVR = distribution volume ratio; MRTM2 = multilinear reference tissue modelling 2; STN = subthalamic nucleus; DBM = dorsal midbrain; DN = dentate nucleus; DLPFC = dorsolateral prefrontal cortex; MPFC = medial prefrontal cortex; m = months; y = years

© 2020 Brendel et al. *JAMA Neurology.*

eTable. Extended study overview

Demographics																		
Study group/ subgroup	**PSP-RS**			**PSP-non-RS**			PSP-CBS	PSP-F	PSP-P	PSP-SL	PSP-PGF	**α-synucleinopathies**	MSA	PD	**Alzheimer's disease**	AD-MCI	AD-Dementia	**Healthy controls**
n	**40**			**20**			9	5	4	1	1	**10**	4	6	**10**	2	8	**10**
Age	**71 ± 6**			**71 ± 9**			70 ± 11	68 ± 4	77 ± 10	72	62	**61 ± 8**	63 ± 6	60 ± 10	**69 ± 10**	72/ 63	69 ± 11	**67 ± 7**
Gender	**18 female / 22 male**			**9 female / 11 male**			6 female/ 3 male	1 female/ 4 male	2 female/ 2 male	1 male	1 male	**3 female / 7 male**	1 female/ 3 male	2 female/ 4 male	**5 female / 5 male**	1 female/ 1 male	4 female/ 4 male	**8 female / 2 male**
Center	**MUC: 21/ LPZ: 11/ COL: 3/ MNI: 4**			**MUC: 16/ LPZ: 4**			MUC: 9	MUC: 3/ LPZ: 2	MUC: 2/ LPZ: 2	MUC: 1	MUC: 1	**MUC: 10**	MUC: 4	MUC: 6	**MUC: 6/ MNI: 4**	MUC: 1/ MNI: 1	MUC: 5/ MNI: 3	**MNI: 5/ AUS: 5**
PSP Rating Scale	**37.2 ± 15.1**			**26.2 ± 9.6**			22 ± 9	30 ± 9	34 ± 7	21	17	**n.a.**	n.a.	n.a.	**n.a.**	n.a.	n.a.	**n.a.**
Possible (n) / Probable (n) PSP	**6 / 34**			**12 / 8**			9 / 0	1 / 4	0 / 4	1 / 0	1 / 0							
Hoehn & Yahr	n.a.			n.a.			n.a.	n.a.	n.a.	n.a.	n.a.	2.4 ± 0.8	2.8 ± 0.5	2.2 ± 1.0	n.a.	n.a.	n.a.	n.a.
UPDRS	n.a.			n.a.			n.a.	n.a.	n.a.	n.a.	n.a.	23.9 ± 6.2	26.0 ± 6.3	22.5 ± 6.3	n.a.	n.a.	n.a.	n.a.
Disease duration (m)	**49 ± 38**			**42 ± 37**			36 ± 21	18 ± 11	64 ± 58	44	95	**20 ± 17**	26 ± 26	17 ± 9	**28 ± 29**	12/ 14	35 ± 31	**n.a.**
MoCA	**20.7 ± 7.5**			**23.1 ± 3.9**			24 ± 4	21 ± 7	24 ± 4	24	20	**25.6 ± 4.1**	23 ± 4	27 ± 4	**15.6 ± 7.8**	27/ 25	13 ± 6	**28.8 ± 1.6**
SEADL	**55 ± 21**			**65 ± 17**			64 ± 15	70 ± 21	58 ± 21	70	60	**n.a.**	n.a.	n.a.	**n.a.**	n.a.	n.a.	**n.a.**
PET results																		
Globus pallidus externus	**1.16 ± 0.10**			**1.10 ± 0.11**			1.08 ± 0.13	1.10 ± 0.10	1.14 ± 0.11	0.99	1.15	**1.01 ± 0.06**	1.01 ± 0.04	1.01 ± 0.07	**1.05 ± 0.06**	1.08/ 1.11	1.04 ± 0.07	**0.99 ± 0.05**
	**$d_{\alpha\text{-}syn}$: 1.83**	**d_{AD}: 1.28**	**d_{HC}: 2.13**	**$d_{\alpha\text{-}syn}$: 0.94**	**d_{AD}: 0.47**	**d_{HC}: 1.20**												
	**$p_{\alpha\text{-}syn}$: .03**	**p_{AD}: .01**	**p_{HC}: <.001**	**$p_{\alpha\text{-}syn}$: >.99**	**p_{AD}: >.99**	**p_{HC}: .02**												

© 2020 Brendel et al. *JAMA Neurology.*

Globus pallidus internus	**1.21 ± 0.10**			**1.12 ± 0.11**			1.10 ± 0.12	1.12 ± 0.09	1.18 ± 0.12	1.01	1.22	**1.03 ± 0.05**	1.02 ± 0.04	1.04 ± 0.05	**1.08 ± 0.06**	1.08/ 1.06	1.08 ± 0.07	**1.00 ± 0.08**
	**$d_{\alpha\text{-syn}}$: 2.23**	**d_{AD}: 1.49**	**d_{HC}: 2.28**	**$d_{\alpha\text{-syn}}$: 1.08**	**d_{AD}: 0.45**	**d_{HC}: 1.27**												
	**$p_{\alpha\text{-syn}}$: .005**	**p_{AD}: .002**	**p_{HC}: <.001**	**$p_{\alpha\text{-syn}}$: >.99**	**p_{AD}: >.99**	**p_{HC}: .009**												
Putamen	**1.19 ± 0.10**			**1.14 ± 0.12**			1.15 ± 0.14	1.15 ± 0.14	1.15 ± 0.11	1.05	1.13	**1.05 ± 0.06**	1.04 ± 0.04	1.06 ± 0.08	**1.10 ± 0.05**	1.12/ 1.16	1.09 ± 0.05	**1.02 ± 0.06**
	**$d_{\alpha\text{-syn}}$: 1.65**	**d_{AD}: 1.12**	**d_{HC}: 2.04**	**$d_{\alpha\text{-syn}}$: 0.93**	**d_{AD}: 0.43**	**d_{HC}: 1.26**												
	**$p_{\alpha\text{-syn}}$: .13**	**p_{AD}: .13**	**p_{HC}: .002**	**$p_{\alpha\text{-syn}}$: >.99**	**p_{AD}: >.99**	**p_{HC}: .10**												
Subthalamic nucleus	**1.21 ± 0.08**			**1.15 ± 0.09**			1.12 ± 0.11	1.16 ± 0.09	1.19 ± 0.06	1.09	1.23	**1.09 ± 0.06**	1.09 ± 0.07	1.09 ± 0.07	**1.10 ± 0.08**	1.16/ 1.16	1.09 ± 0.08	**1.04 ± 0.09**
	**$d_{\alpha\text{-syn}}$: 1.67**	**d_{AD}: 1.37**	**d_{HC}: 2.02**	**$d_{\alpha\text{-syn}}$: 0.80**	**d_{AD}: 0.59**	**d_{HC}: 1.26**												
	**$p_{\alpha\text{-syn}}$: .06**	**p_{AD}: .003**	**p_{HC}: <.001**	**$p_{\alpha\text{-syn}}$: >.99**	**p_{AD}: .64**	**p_{HC}: .005**												
Substantia nigra	**1.17 ± 0.09**			**1.13 ± 0.09**			1.12 ± 0.10	1.13 ± 0.11	1.14 ± 0.06	1.05	1.23	**1.09 ± 0.06**	1.05 ± 0.06	1.12 ± 0.04	**1.12 ± 0.08**	1.16/ 1.11	1.11 ± 0.09	**1.10 ± 0.07**
	**$d_{\alpha\text{-syn}}$: 1.08**	**d_{AD}: 0.64**	**d_{HC}: 0.89**	**$d_{\alpha\text{-syn}}$: 0.50**	**d_{AD}: 0.14**	**d_{HC}: 0.35**												
	**$p_{\alpha\text{-syn}}$: >.99**	**p_{AD}: .70**	**p_{HC}: .04**	**$p_{\alpha\text{-syn}}$: >.99**	**p_{AD}: >.99**	**p_{HC}: .53**												
Dorsal midbrain	**0.87 ± 0.11**			**0.89 ± 0.09**			0.87 ± 0.09	0.94 ± 0.10	0.88 ± 0.08	0.90	0.88	**0.92 ± 0.07**	0.86 ± 0.06	0.95 ± 0.04	**0.93 ± 0.09**	0.97/ 0.84	0.93 ± 0.09	**0.92 ± 0.10**
	**$d_{\alpha\text{-syn}}$: -0.50**	**d_{AD}: -0.58**	**d_{HC}: -0.45**	**$d_{\alpha\text{-syn}}$: -**	**d_{AD}: -0.43**	**$d_{\alpha\text{-syn}}$: -**												

© 2020 Brendel et al. *JAMA Neurology.*

				**0.32**		**0.28**												
	**$p_{\alpha\text{-syn}}$: >.99**	**p_{AD}: >.99**	**p_{HC}: >.99**	**$p_{\alpha\text{-syn}}$: >.99**	**p_{AD}: >.99**	**$p_{\alpha\text{-syn}}$: >.99**												
Dentate nucleus	**1.13 ± 0.05**			**1.11 ± 0.05**			1.10 ± 0.05	1.13 ± 0.04	1.15 ± 0.07	1.06	1.10	**1.07 ± 0.05**	1.09 ± 0.03	1.06 ± 0.06	**1.08 ± 0.03**	1.11/ 1.04	1.09 ± 0.03	**1.06 ± 0.04**
	**$d_{\alpha\text{-syn}}$: 1.26**	**d_{AD}: 1.17**	**d_{HC}: 1.68**	**$d_{\alpha\text{-syn}}$: 0.84**	**d_{AD}: 0.67**	**d_{HC}: 1.18**												
	**$p_{\alpha\text{-syn}}$: .13**	**p_{AD}: .08**	**p_{HC}: .03**	**$p_{\alpha\text{-syn}}$: >.99**	**p_{AD}: >.99**	**p_{HC}: .41**												
Dorsolateral prefrontal cortex	**0.89 ± 0.09**			**0.91 ± 0.06**			0.92 ± 0.07	0.91 ± 0.07	0.91 ± 0.04	0.88	0.94	**0.91 ± 0.05**	0.95 ± 0.05	0.88 ± 0.04	**1.11 ± 0.24**	0.99/ 0.94	1.15 ± 0.25	**0.86 ± 0.08**
	**$d_{\alpha\text{-syn}}$: -0.28**	**d_{AD}: -1.24**	**d_{HC}: 0.28**	**$d_{\alpha\text{-syn}}$: 0.09**	**d_{AD}: -1.13**	**d_{HC}: 0.70**												
	**$p_{\alpha\text{-syn}}$: >.99**	**p_{AD}: <.001**	**p_{HC}: >.99**	**$p_{\alpha\text{-syn}}$: >.99**	**p_{AD}: <.001**	**p_{HC}: >.99**												
Medial prefrontal cortex	**0.83 ± 0.09**			**0.86 ± 0.09**			0.87 ± 0.10	0.82 ± 0.07	0.87 ± 0.07	0.76	0.96	**0.89 ± 0.06**	0.91 ± 0.09	0.88 ± 0.05	**0.98 ± 0.12**	0.95/ 0.89	0.99 ± 0.14	**0.89 ± 0.08**
	**$d_{\alpha\text{-syn}}$: -0.77**	**d_{AD}: -1.39**	**d_{HC}: -0.74**	**$d_{\alpha\text{-syn}}$: -0.41**	**d_{AD}: -1.14**	**d_{HC}: -0.42**												
	**$p_{\alpha\text{-syn}}$: .91**	**p_{AD}: <.001**	**p_{HC}: >.99**	**$p_{\alpha\text{-syn}}$: >.99**	**p_{AD}: .008**	**p_{HC}: >.99**												

Demographics and quantitative PET results of main and subgroups of the study. P values were derived from MANOVA including center, age and gender as covariates and Bonferroni adjustment for multiple comparisons of study groups. Effect sizes were calculated as Cohens d (d) for both PSP study groups against different control groups. PSP = Progressive supranuclear palsy; RS = Richardson syndrome; MV = mean value; SD = standard deviation; DVR = distribution volume ratio; m = months; AD = Alzheimer's disease; HC = healthy controls; α-syn = probable α-synucleinopathies; n = sample size; UPDRS = Unified Parkinson's Disease Rating Scale; MoCA = Montreal Cognitive Assessment; SEADL = Schwab and England Activities of Daily Living; n.a. = not available; MUC = scan site Munich, Germany; LPZ = scan site Leipzig, Germany; COL = scan site Cologne, Germany; MNI = scan site New Haven, US; AUS = scan site Melbourne, Australia; CBS = PSP with predominant corticobasal syndrome; SL = PSP with predominant speech/language impairment; F = PSP with predominant frontal presentation; P = PSP with predominant Parkinsonism; PGF = PSP with progressive gait freezing; PD = Parkinson's disease; MSA = multiple system atrophy; AD-MCI = mild cognitive impairment due to AD

© 2020 Brendel et al. *JAMA Neurology.*
